# Exploring Magnetic Exchange Coupling: Synthesis and Characterization of Magnetite-Based Composites

**DOI:** 10.1021/acs.jchemed.5c01804

**Published:** 2026-02-19

**Authors:** Mostafa G. Mohamed, James Lambe, Kenneth Hernandez, Carlos Blank, Camilo Bedoya López, Carlos E. Castano

**Affiliations:** 1 Department of Mechanical and Nuclear Engineering, 6889 Virginia Commonwealth University, Richmond, Virginia 23284, United States; 2 Polymers and Pigments Department, 68787 National Research Centre, Dokki, Cairo Egypt; 3 Rose-Hulman Institute of Technology, Terre Haute, Indiana 47803, United States

**Keywords:** Biomedical applications, Magnetic exchange coupling, Environmental remediation

## Abstract

This laboratory experiment is designed for Research Experiences for Undergraduates (REU) programs, offering students immersive, hands-on research opportunities in the synthesis and characterization of magnetic materials. It emphasizes the foundational principles of magnetism, explores the essential properties of magnetic materials, and introduces various characterization techniques. The protocol highlights the significance of magnetite-based materials in diverse applications, providing a focused investigation into magnetic exchange coupling and enabling students to connect fundamental magnetic phenomena with cutting-edge research. Students conduct four experiments to prepare magnetite-based composites that incorporate both titanium and cobalt oxides. This approach allows them to explore magnetic exchange coupling and examine the resulting magnetic properties. By combining magnetite (Fe_3_O_4_), a well-known magnetic material, with titanium dioxide (TiO_2_), a diamagnetic oxide, and cobalt ferrite (CoFe_2_O_4_), a strong ferrimagnetic oxide with high coercivity, students investigate how the interaction between soft and hard magnetic phases affects overall magnetization behavior and magnetic coupling efficiency. Students then characterize these composites using techniques such as X-ray diffraction and vibrating sample magnetometry to study their magnetic properties and chemical structure, deepening their understanding of how these factors influence material behavior. This integrated approach reinforces core concepts of magnetism, materials science, and engineering while equipping students with practical skills in material preparation and characterization.

## Graphical abstract

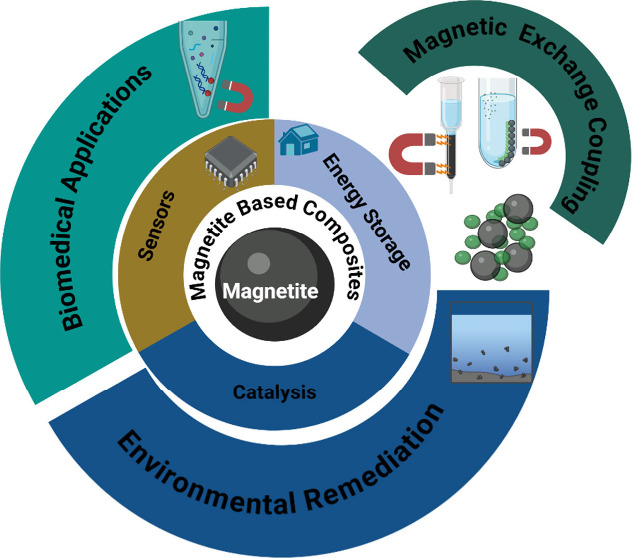

## 1 Introduction/Background

A solid foundation in materials science is essential for undergraduate engineering students, as it bridges the gap between fundamental scientific principles and real-world applications. Understanding how materials function, how they are prepared, and how their properties can be tailored is essential for driving innovation in various engineering fields, including electronics, energy, and biomedical engineering.^1^

Magnetic materials, especially those based on magnetite (Fe_3_O_4_), are of particular importance in engineering. Magnetite’s unique properties of high saturation magnetization, electrical conductivity, and chemical stability make it valuable for applications such as sensors, data storage, spintronics, and electromagnetic shielding.^2–5^ Its magneto-crystalline anisotropy arising from the specific arrangement and coupling of iron cations in the crystal lattice enable advanced functionalities in devices that rely on the control and manipulation of magnetic fields.^6,7^ For instance, in spintronic devices, the manipulation of electron spin (rather than charge) relies heavily on the magnetic coupling and polarization properties of materials like magnetite.^8^

The concept of magnetic coupling exchange, including the interaction between magnetic moments in composite or multiphase materials, is essential to the design of next-generation magnetic devices. By coupling magnetite with other materials with different magnetic behavior, it is possible to alter and enhance key properties, including coercivity, remanence, and overall magnetic response. Such modifications are essential for optimizing materials for specific engineering functions, including high-density data storage, efficient electromagnetic sensors, and robust actuators.^9–11^

A review of the educational literature reveals that many previous studies have highlighted the value of magnetic materials in undergraduate education, often focusing on experimental protocols for material preparation. These protocols typically integrate chemical synthesis methods, such as coprecipitation, sol–gel, and hydrothermal methods, enabling students to gain hands-on experience in preparing magnetic materials and exploring how preparation conditions affect their properties.^12–14^ However, a standard limitation of these studies is the lack of emphasis on the magnetic exchange coupling concept and the characterization techniques used in this area, such as Vibrating Sample Magnetometry (VSM), which are essential for understanding and quantifying the magnetic behavior of materials. In particular, the hysteresis loop, which is a fundamental concept in magnetism that describes the relationship between magnetization and applied magnetic field, remains underexplored in many undergraduate experiments, despite its critical importance for interpreting material performance and characteristics in real-world applications. Additionally, X-ray diffraction (XRD) can be used as an essential characterization tool for defining and monitoring changes in material phases during preparation and modification. Powder X-ray diffraction can be found in literature with the acronym PXRD to distinguish from single crystal XRD, but it is often simply referred to just as XRD. As a reference, XRD in this work will represent powder X-ray diffraction. As XRD provides detailed information about the crystalline structure, it allows students to learn how to identify and prove the formation of desired phases or detect new ones resulting from different preparation methods and during the coupling processes. This insight is crucial for correlating structural changes with observed magnetic or functional properties, and for ensuring the success of the chemical preparation techniques employed.

In conclusion, this laboratory protocol aims to provide students with direct experience in both the chemical preparation and magnetic coupling of magnetite-based materials. Using coprecipitation, students prepare magnetite and couple it with TiO_2_ and cobalt ferrite, observing how these couplings influence the resulting magnetic properties. For example, Fe_3_O_4_/TiO_2_ coupling may lead to changes in surface properties and potentially reduce saturation magnetization due to the nonmagnetic nature of TiO_2_,^15^ but could enhance photocatalytic or electronic functionalities. Meanwhile, magnetite-cobalt ferrite Fe_3_O_4_/CoFe_2_O_4_ coupling is expected to increase coercivity and magnetic anisotropy, as cobalt ferrite is a hard magnetic material, potentially resulting in a composite with improved magnetic stability and tunable properties suitable for advanced engineering applications.^16^

Through this integrated approach, students should not only learn the preparation techniques but also gain insight into the critical role of magnetic exchange coupling and the necessity of characterization methods such as VSM and XRD in the development and optimization of magnetic materials for engineering innovation.

## 2 Experimental Work

This experiment series is designed for Research Experience for Undergraduate Program (REU) sophomores or juniors with a materials science and engineering background, comprising five weekly 3-h sessions. REUs are competitive research programs that allow undergraduate students to immerse themselves in ongoing research-funded projects over the summer; however, it is often challenging to become proficient in synthesis and characterization within such a short period without guidance, and this work summarizes our best practices after years of experience onboarding undergraduate students in summer experiences such as REUs. In the first four sessions, one-third of the time is dedicated to theoretical background and scientific fundamentals, with the remaining time devoted to practical and experimental work. In the final session, the student groups (2–3 students) should present their results and discuss them with instructors as part of the research experience. The flowchart of the work to be conducted over the 5 weeks is presented in Figure 1.

**Figure 1:**
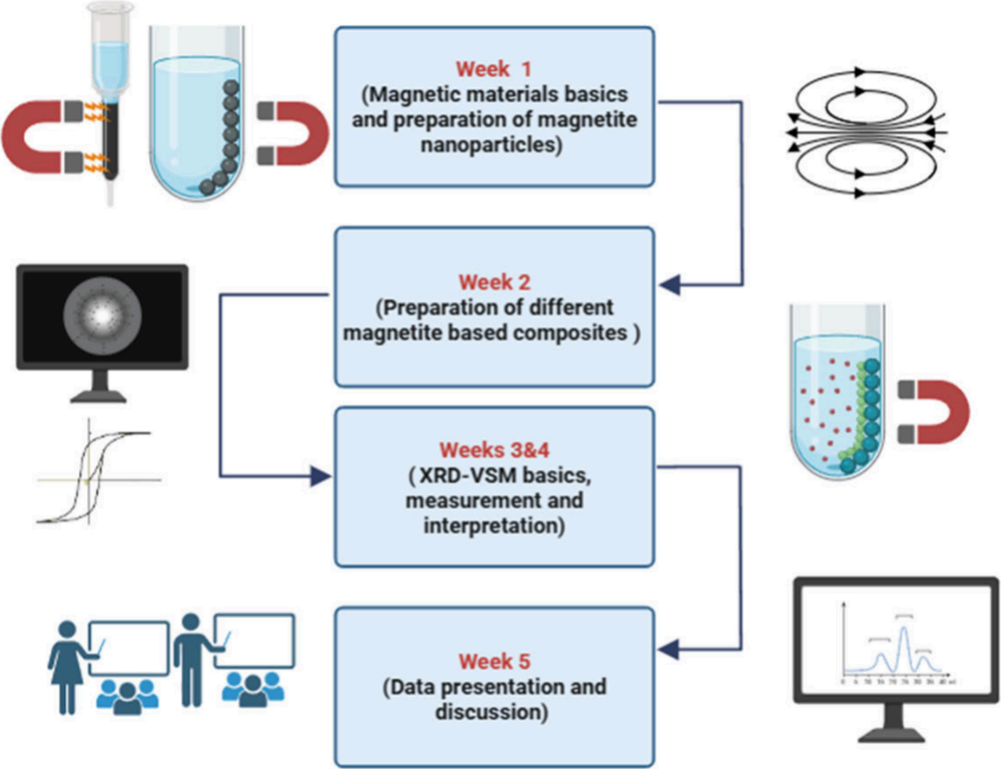
Flowchart outlining the experimental work to be conducted over the 5 weeks.

### Week One: Introduction and Magnetite Preparation

The first session opens with a detailed overview of the fundamental concepts of magnetic materials, focusing on the various types of magnetism such as paramagnetism, diamagnetism, ferromagnetism, Antiferromagnetism and Ferrimagnetism, see Figure 2. Real-world examples are discussed to illustrate how these magnetic behaviors manifest in various materials, thereby establishing a strong conceptual foundation for the experimental work that follows.

**Figure 2:**
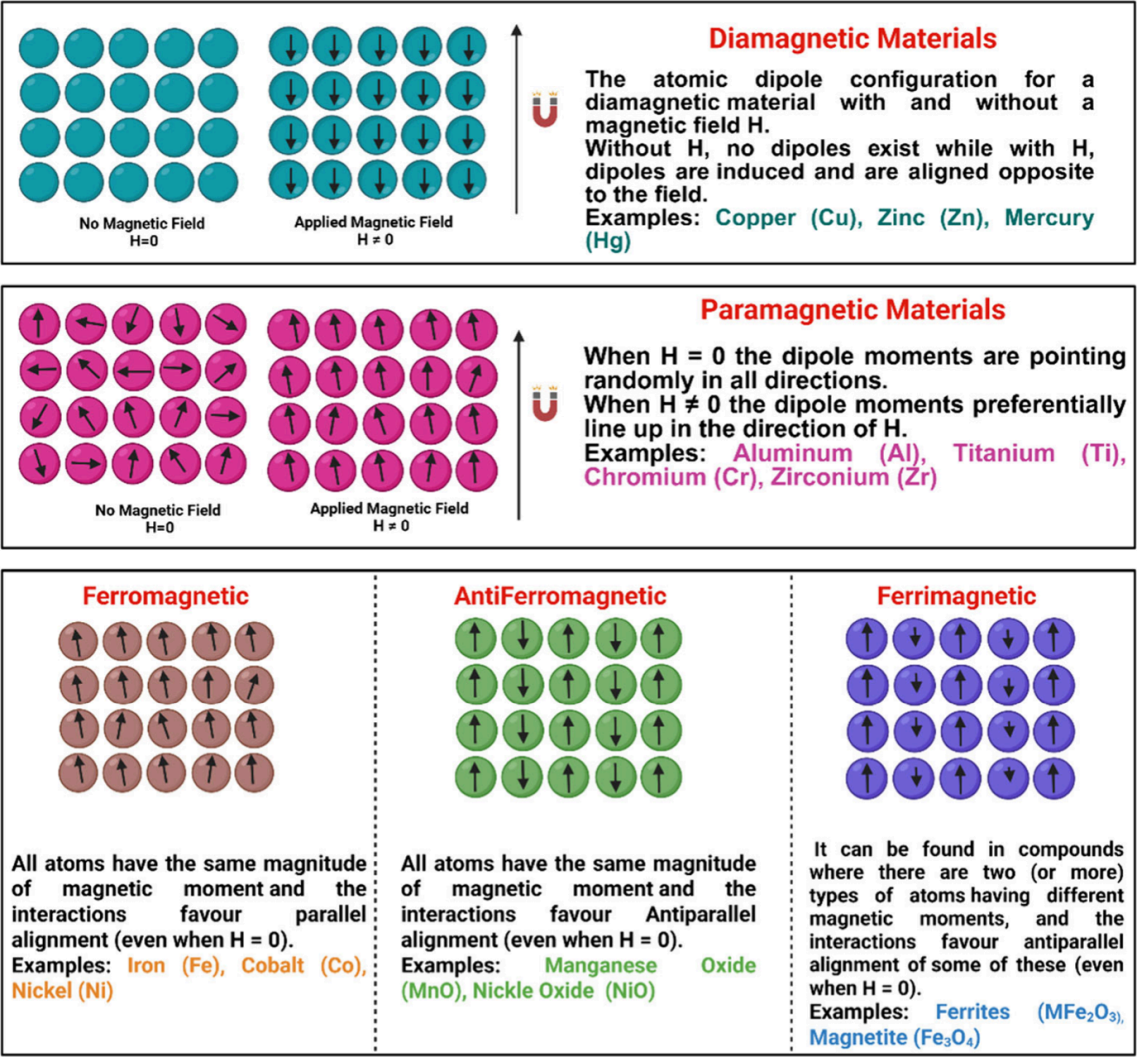
Illustration depicting different types of magnetic materials.

Following this, a thorough explanation of the experimental workflow is provided to ensure all students understand each step of the process. The experiment for the first week focuses on the preparation of nanostructured magnetite (Fe_3_O_4_) using the coprecipitation method, a technique extensively described in scientific literature. The procedure involves the controlled coprecipitation of iron salts under alkaline conditions, resulting in the formation of uniform magnetite nanoparticles. The coprecipitation method for preparing magnetite (Fe_3_O_4_) nanoparticles involves dissolving iron­(III) chloride hexahydrate (FeCl_3_·6H_2_O) and iron­(II) sulfate heptahydrate (FeSO_4_·7H_2_O) in deionized water, typically maintaining a Fe^3+^:Fe^2+^ molar ratio of 2:1 (100 mL of 0.2 mol L^–1^ FeCl_3_·6H_2_O and 100 mL of 0.1 mol L^–1^ FeSO_4_·7H_2_O), as widely reported in the literature.^17–21^ The mixed iron solution is heated to 60 °C with constant stirring for 15 min, and a 2 mol L^–1^ sodium hydroxide (NaOH) solution is added dropwise until the pH reaches 10–11, resulting in the immediate formation of a black magnetite precipitate. Students can observe the formation of magnetite nanoparticles by positioning a strong magnet near the reaction beaker as presented in Figure 3.

**Figure 3:**
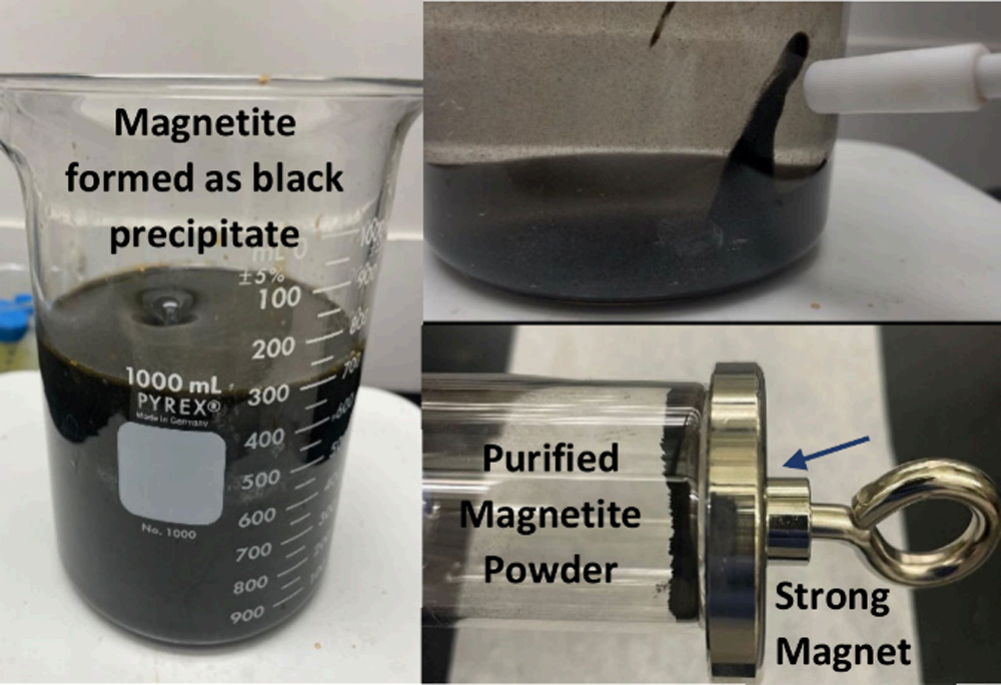
Preparation of magnetite nanoparticles.

The mixture is then stirred at the same temperature for 30 min to ensure complete reaction and particle growth. The synthesized nanoparticles were first allowed to cool, then transferred into centrifuge tubes and centrifuged at 5000 rpm for 10 min. The supernatant was carefully decanted to separate the nanoparticle pellet. This was followed by two successive washes with deionized water to effectively remove any residual ions or impurities. Finally, the purified magnetite nanoparticles were dried under vacuum at 40 °C to remove moisture while preserving their structural integrity. A comprehensive description of the experimental procedure and the materials employed is provided in the Supporting Information (Experiment 1).

In this experiment, Fe_3_O_4_ nanoparticles were synthesized via coprecipitation method to allow students to learn about reaction kinetics, pH management, and thermal control during the procedure. Alternatively, equivalent magnetite nanoparticles can be prepared more rapidly at room temperature using FeCl_2_ with dilute NH_3_, as reported by (Dalverny et al. 2017).^22^

### Week Two: Magnetite Composite Preparation

The second session focuses on the basics of magnetic coupling, discussing how the procedures and the resulting chemical structure can affect the magnetic properties of the materials produced, with particular attention to the differences between soft, hard and nonmagnetic phases (Supporting Information: Experiment 2).

During the experimental session, students should be divided into two groups. Each group is responsible for conducting experiments on specific magnetic couplings: one group can work with titanium dioxide, while the other focuses on cobalt ferrite.

Students take the magnetite nanoparticles prepared in the previous session and begin coupling them with titanium dioxide (TiO_2_) and cobalt ferrite (CoFe_2_O_4_), gaining hands-on experience with composite magnetic materials (schematic diagram for the preparation procedure can be found in Figure 4). The process starts with a pretreatment step,^20,23^ where the magnetite nanoparticles are dispersed in a citric acid solution and sonicated for 10 min, allowing citric acid to chemisorb onto the surface and enhance the dispersion and stability of the particles. In this step the students learn about the surface modification and the concept of pretreatment for the heterogeneous nucleation of the titanium dioxide and ferrite on the magnetite as secondary phase. For the preparation of Fe_3_O_4_/TiO_2_ composite,^24,25^ the citric acid-modified magnetite nanoparticles are first suspended in deionized water. Separately, titanium tetrachloride (TiCl_4_) is hydrolyzed in dilute HCl solution to form a stable TiOCl_2_ precursor, which is then added dropwise to the magnetite suspension under controlled temperature and pH conditions. Hydrolysis and TiO_2_ formation on the presence of magnetite are completed by subsequent dropwise addition of NaOH solution to reach pH 10–12, followed by heating and stirring. The addition of TiCl_4_ should be carried out only under the direct supervision of senior researchers or instructors, as titanium tetrachloride is highly fuming and extremely corrosive. Through this procedure, in addition to the scientific concept of the magnetic exchange coupling and preparation method, students are expected to gain practical training in the safe handling of volatile, moisture-sensitive reagents (like TiCl_4_) and in their controlled introduction into a reaction system. However, alternative precursors to TiCl_4_ (e.g., titanium­(IV) ethoxide (TEOT), titanium­(IV) isopropoxide (TTIP), and titanium­(IV) butoxide (TBT)) can be used with some modification on the procedure to achieve the same composite structure with fewer safety considerations.^26,27^

**Figure 4:**
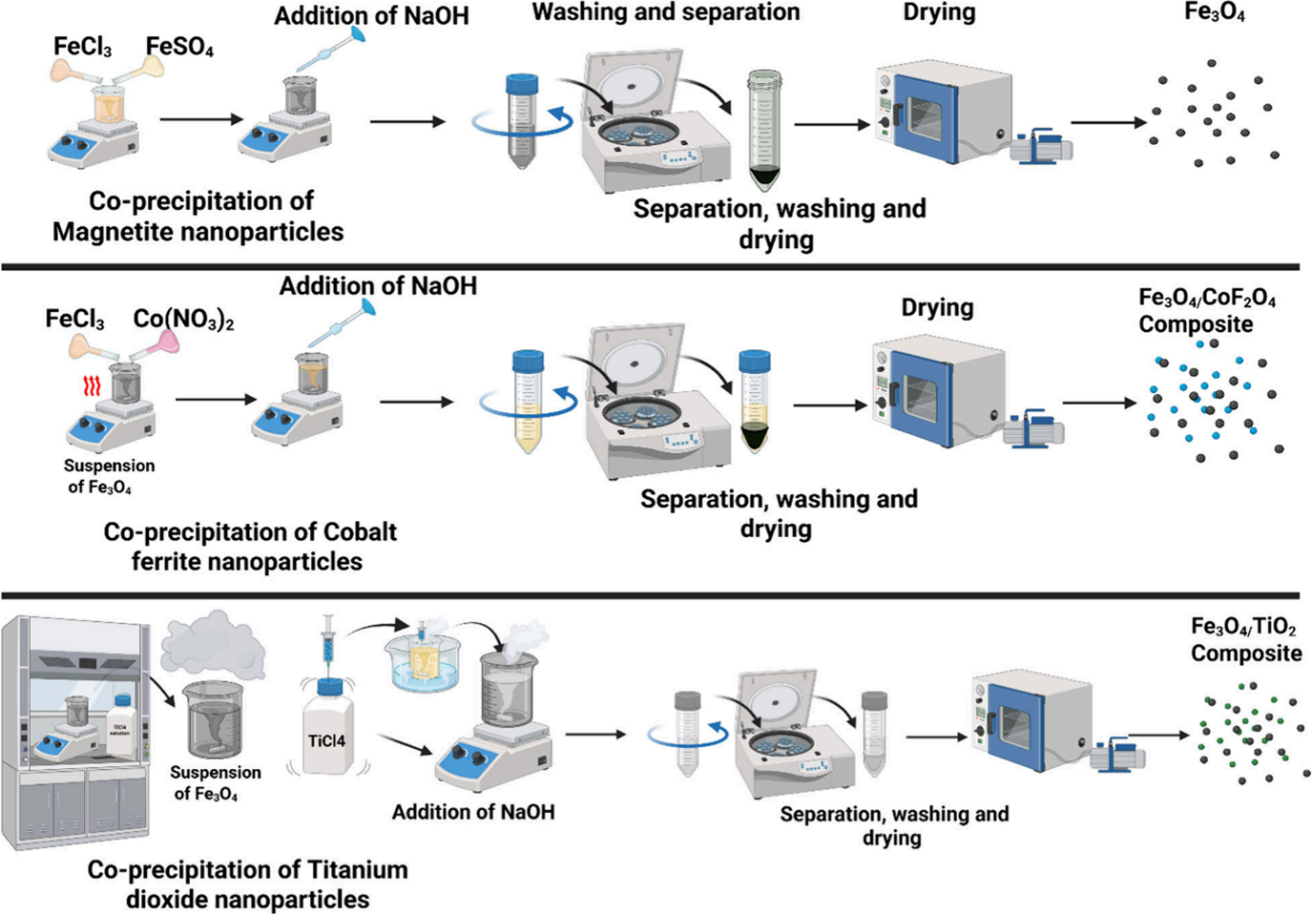
Schematic representation of the preparation procedures for the various.

For the preparation of Fe_3_O_4_/CoFe_2_O_4_ composites,^28,29^ cobalt nitrate hexahydrate, and iron­(III) chloride hexahydrate are dissolved in water and added to the pretreated magnetite suspension, followed by the gradual addition of NaOH to induce coprecipitation of cobalt ferrite onto the magnetite, again under controlled temperature and stirring. The full procedures with the quantities used can be found in (Supporting Information: ).

By the end of this session, students should have developed practical competence in weighing reagents, preparing solutions of different concentrations, performing stoichiometric and concentration calculations, and setting up synthetic procedures. They should also gain experience in the safe handling of reagents with special characteristics, such as TiCl_4_, while simultaneously deepening their theoretical understanding of magnetic behavior and the role of magnetic coupling in determining material properties.

### Week Three/Four: Materials Characterization

In week three, the theoretical part begins with a recap and a focused overview of X-ray diffraction (XRD). The instructor explains how XRD patterns are interpreted to distinguish between different phases present in the samples and to confirm the successful formation of composite structures such as Fe_3_O_4_/TiO_2_ and Fe_3_O_4_/CoFe_2_O_4_. Following this in Week four, the basics of vibrating sample magnetometry (VSM) will be introduced, covering the fundamental principles of magnetic measurements, hysteresis loop basics, and key parameters such as saturation magnetization, coercivity, and remanence, and how these relate to the soft and hard magnetic properties discussed in previous sessions (Supporting Information: Experiments 3 and 4).

Following the theoretical discussion, students move on to the practical part, where they analyze samples prepared in previous weeks using both XRD and VSM techniques. The instructors should conduct a live demonstration of the proper procedures for sample preparation and loading for both instruments. Groups rotate between the two devices, and due to the limitations of these devices and the long time for each sample, graduate students/postdocs operate the instruments and run all samples collected from the various student groups, ensuring thorough characterization of each group’s materials. The results then should be returned to the students to facilitate data plotting and interpretation.

### Week Five: Presentation and Discussion

In the final week, students should be previously informed to plot their data for both XRD and VSM analyses. Each group is required to explain the XRD results in the context of phase analysis, highlighting the differences between magnetite and its coupling with other materials. For the VSM analysis, each group presents the hysteresis loop of magnetite coupled with one of the materials, comparing key parameters such as saturation and coercivity. They should also explain how coupling affects the magnetic properties of the materials and discuss potential applications. All data must be presented in slides, accompanied by explanations.

## 3 Hazard and Safety

Before beginning the program, all students should complete comprehensive safety training covering the correct use of personal protective equipment (PPE), emergency response protocols (including the locations and operation of eye-wash stations and chemical showers), in addition to the identification and classification of chemical hazards.

All procedures were performed in a certified fume hood to minimize exposure to harmful vapors. Standard PPE, including lab coats, chemical-resistant goggles, and appropriate gloves (nitrile), should be always worn.

When handling powders, students should work in a well-controlled environment that minimizes dust formation and airborne dispersion, such as a fume hood or enclosed balance area. They must wear full personal protective equipment, including a lab coat, appropriate gloves, safety goggles, and a suitable dust mask or respirator to reduce inhalation and contact risks.

Some chemicals used in these experiments, such as cobalt compounds (e.g., Co­(NO_3_)_2_), are toxic with chronic exposure, while others like NaOH is highly corrosive to skin, eyes, and respiratory tract. So, these materials should be handled in a fume hood with nitrile gloves, safety goggles, and lab coat.

In this experimental program, one of the aims is to give students supervised, hands-on experience with a reagent that demands special care. All operations involving TiCl_4_ are done under the direct supervision of senior researchers or the instructors, who guide the students through safe transfer, controlled addition to the magnetite suspension, and correct disposal of TiCl_4_-containing waste (The specific precautions of handling the TiCl_4_ is mentioned in details in the Safety notes after the procedures (Supporting Information, ).

## 4 Results and Discussion

### 4.1 Powder X-ray Diffraction (XRD)

After preparing the magnetite and coupling it with titanium dioxide and cobalt ferrite, the next step involves analyzing the prepared materials. Each group should receive the raw XRD data along with various powder diffraction files to plot the XRD results and conduct the necessary interpretations. Students are expected to carefully examine the provided XRD patterns by matching the observed diffraction peaks with standard reference patterns to confirm the formation of the targeted composites Fe_3_O_4_/CoFe_2_O_4_ and Fe_3_O_4_/TiO_2_ by verifying the coexistence of characteristic peaks from both magnetite and the secondary phase (cobalt ferrite or titanium dioxide) and assessing the crystallinity of the samples.

Figure 5 shows the XRD results for the prepared materials, obtained through the coprecipitation method in the synthesis of magnetite and its coupling with titanium dioxide and cobalt ferrite. In this section, we provide a template for reporting XRD results to share with students after they present their data, demonstrating best practices in reporting such characterization data. For the Fe_3_O_4_/CoFe_2_O_4_ composite, as both Fe_3_O_4_ and CoFe_2_O_4_ have cubic spinel structures with nearly identical lattice parameters, leading to very similar XRD patterns where main reflections such as (220), (311), (400), (422), (511), and (440) almost exactly overlap. Previous studies showed that XRD alone cannot reliably distinguish between magnetite and cobalt ferrite composites structures, especially with small percentages of cobalt added to the structure.^30–32^ In analyzing the XRD of these composites, students should learn how the XRD of materials with similar crystal structures and chemical compositions may show overlapping diffraction patterns. Therefore, researchers at various stages of their research need to use complementary characterization techniques to verify their theory and prove their material’s structure. Additionally, while XRD may reveal no significant differences, students observe clear distinctions in the magnetic parameters through VSM measurements. On the other hand, the XRD pattern for the Fe_3_O_4_/TiO_2_ composite clearly displays the characteristic Fe_3_O_4_ peaks alongside prominent peaks (101), (004), (200)) that match the anatase phase of TiO_2_. It is also observed that the broadening of the anatase peaks can be attributed primarily to the nanoscale crystallite size of the anatase phase and the presence of lattice strain. When TiO_2_ exists as nanocrystals, the reduced coherence length of atomic planes leads to a significant broadening of diffraction peaks, as described by the Scherrer equation. Crystallite sizes below about 10–20 nm are particularly associated with broadened XRD reflections.^15,33^

**Figure 5:**
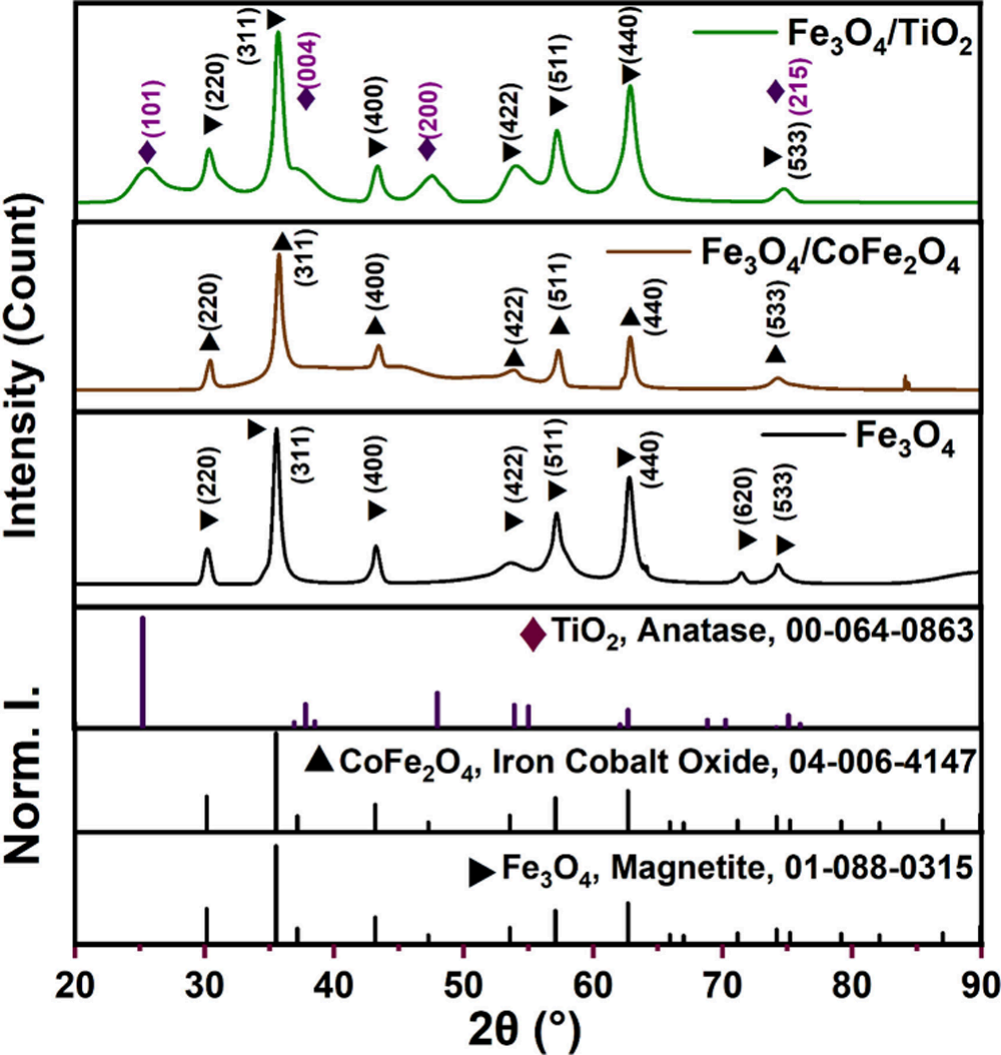
XRD of magnetite and the prepared composites.

### 4.2 Vibrating Sample Magnetometry (VSM)

The vibrating sample magnetometry (VSM) results for the prepared magnetite and composites are presented in Figure 6 and Table 1. The aim of this section is for students to plot their data to learn about the hysteresis loop and begin extracting various parameters, including coercivity (H_c_), saturation magnetization (M_s_), and retentivity (M_r_).

**Table 1:** Saturation Magnetization (M_s_), Retentivity (M_r_), and Coercivity (H_C_) of the Prepared Materials

Material	Saturation Magnetization (emu/g)	Coercivity (Oe)	Retentivity (emu/g)
Fe_3_O_4_	63.31	16.44	1.72
Fe_3_O_4_/TiO_2_	12.01	14.71	0.37
Fe_3_O_4_/CoFe_2_O_4_	43.93	264.1	5.2

**Figure 6:**
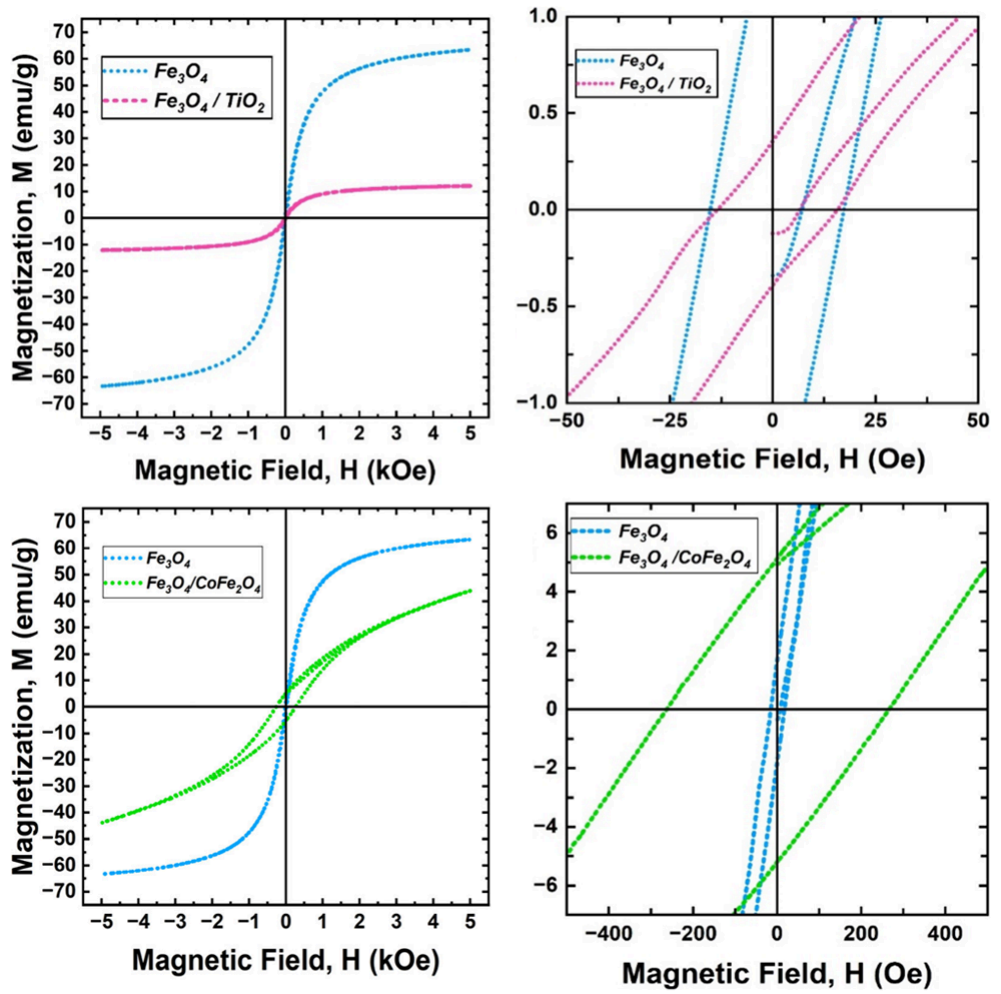
Hysteresis loop of Fe_3_O_4,_ Fe_3_O_4_/TiO_2_, and Fe_3_O_4_/CoFe_2_O_4_.

The prepared magnetite nanomaterial exhibits a hysteresis loop with a relatively steep approach to saturation and a well-defined narrow opening, characteristic of a soft single-domain or near-single-domain system. The corresponding saturation magnetization (M_s_) of 63.31 emu g^–1^ is consistent with literature values for Fe_3_O_4_ nanoparticles with an average size around 30 nm. This estimation is further supported by the coercivity (H_c_) value of 16.44 Oe, aligning with established trends showing that coercivity increases with particle size up to the single-domain limit, then decreases as particles transition into multidomain structures. The retentivity (M_r_), measured at 1.72 emu/g, also supports the ∼ 30 nm size estimation, as it follows a similar trend to the coercivity of single-domain particles, which tend to retain magnetization more effectively than multidomain ones due to the absence of competing magnetic domains.^34–36^ In this section, one of the important targets is relating the shape of the hysteresis loop and the change in the magnetic properties with the particle size and learning about the critical sizes as can be found in the (Supporting Information, ).

When analyzing the magnetic behavior of the prepared composites, a significant reduction in saturation magnetization was observed, which aligns the literature on nanostructures involving nonmagnetic or weak magnetic material. In the Fe_3_O_4_/TiO_2_ composite, the saturation magnetization (M_s_) dropped from 63.31 emu/g in the pure magnetite sample to 12.01 emu/g, indicating substantial magnetic suppression. This pronounced decrease can be attributed to electron migration and spin disorder at the interface of the two materials, where charge transfer into the TiO_2_ disrupts the alignment of magnetic spins in the magnetite. This phenomenon, commonly referred to as the magnetic dead layer or charge-transfer-induced magnetization suppression, effectively reduces the net magnetization reference as can be found in the illustration in Figure 7. Because this dead layer mainly affects how many spins can align rather than how strongly they are pinned, the impact on coercivity (H_c_) is modest, as reflected by the small decrease to 14.71 Oe that still reflects a very soft magnetic system. Coercivity (H_c_) in such composites is governed more by effective anisotropy and domain-wall pinning than by the mere loss of saturated spins, so a disordered surface does not dramatically harden the material. In contrast, retentivity (M_r_) is very sensitive to the fraction of coherently aligned spins; once the field is removed, the interfacial spin disorder and reduced interparticle interactions allow the magnetization to relax more easily to near-zero, which explains the low remanence value of 0.37 emu/g.^36–38^

**Figure 7:**
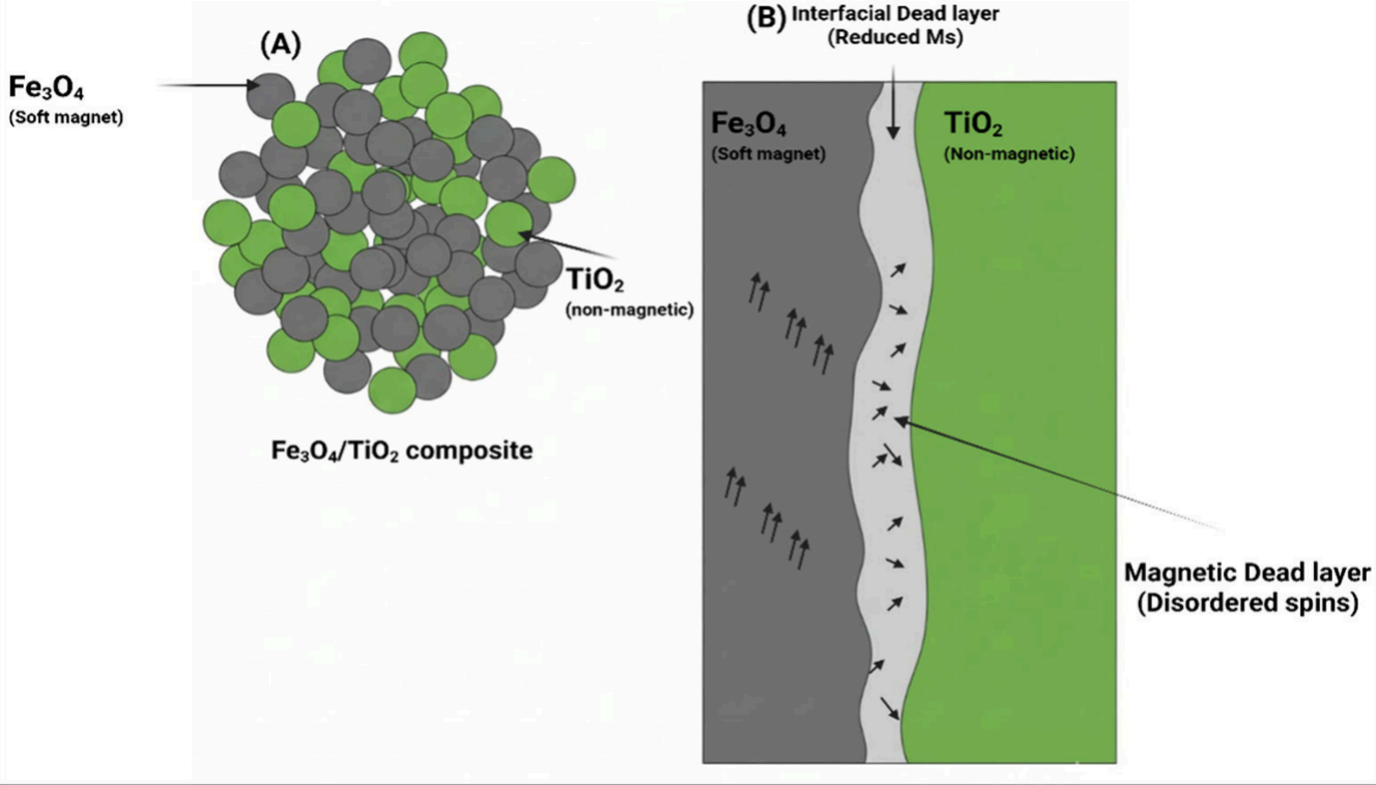
Schematic illustration of dead layer formation at the Fe_3_O_4_/TiO_2_ interface: (a) Fe_3_O_4_ and TiO_2_ nanoparticles in the composite structure; and (b) an interfacial dead layer.

The Fe_3_O_4_/CoFe_2_O_4_ composite shows the typical fingerprint of a soft–hard exchange-coupled system, where the hard CoFe_2_O_4_ strongly modifies the reversal of the soft Fe_3_O_4_. The saturation magnetization (M_s_) of 43.93 emu/g is lower than that of pure Fe_3_O_4_ because part of the composite mass is the cobalt ferrite shell, whose intrinsic M_s_ is lower than magnetite, and because some interfacial spins become canted or frustrated rather than fully aligned. Nevertheless, the remaining magnetically active Fe_3_O_4_ still contributes substantially, so the decrease in M_s_ is moderate rather than drastic, unlike the case of the Fe_3_O_4_/TiO_2_ composite.

On the other hand, what changes most dramatically is the hysteresis shape: the coercivity (H_c_) increases to 264.1 Oe and the retentivity (M_r_) to 5.2 emu/g, indicating a much harder and more magnetically stable material. Magnetic anisotropy means that a material’s magnetization prefers specific directions, creating an energy barrier that resists spin reorientation. Because CoFe_2_O_4_ has high magnetocrystalline anisotropy, it acts as a rigid anchor for the spins at the interface and, when coupled to the lower-anisotropy Fe_3_O_4_ phase, it strongly controls the overall switching behavior, the energy barrier for magnetization reversal, and the efficiency of exchange coupling between the two phases. Through this strong exchange coupling, the soft Fe_3_O_4_ moments near the interface are forced to rotate coherently with the hard CoFe_2_O_4_ shell, so reversing the net magnetization now requires overcoming the anisotropy barriers of both components. Consequently, domain-wall motion and spin rotation are more strongly hindered, which directly leads to the observed increase in coercivity (H_c_) and the larger remanent magnetization (M_r_) after removal of the external field.

From a microstructural perspective and to get the idea of the exchange more imaginable to the students, the system can be viewed as an assumption of exchange-spring magnet on the nanoscale as found in the illustration Figure 8. Under an applied field, the soft Fe_3_O_4_ aligns easily and helps pull the harder CoFe_2_O_4_ shell toward saturation, contributing to a relatively high saturation magnetization (M_s_) for a hard/soft mixture. During reversal, the outer shell resists switching and exerts a restoring torque on the core spins, delaying their reversal and giving rise to the enhanced loop squareness and magnetic hardness you observe.^39,40^

**Figure 8:**
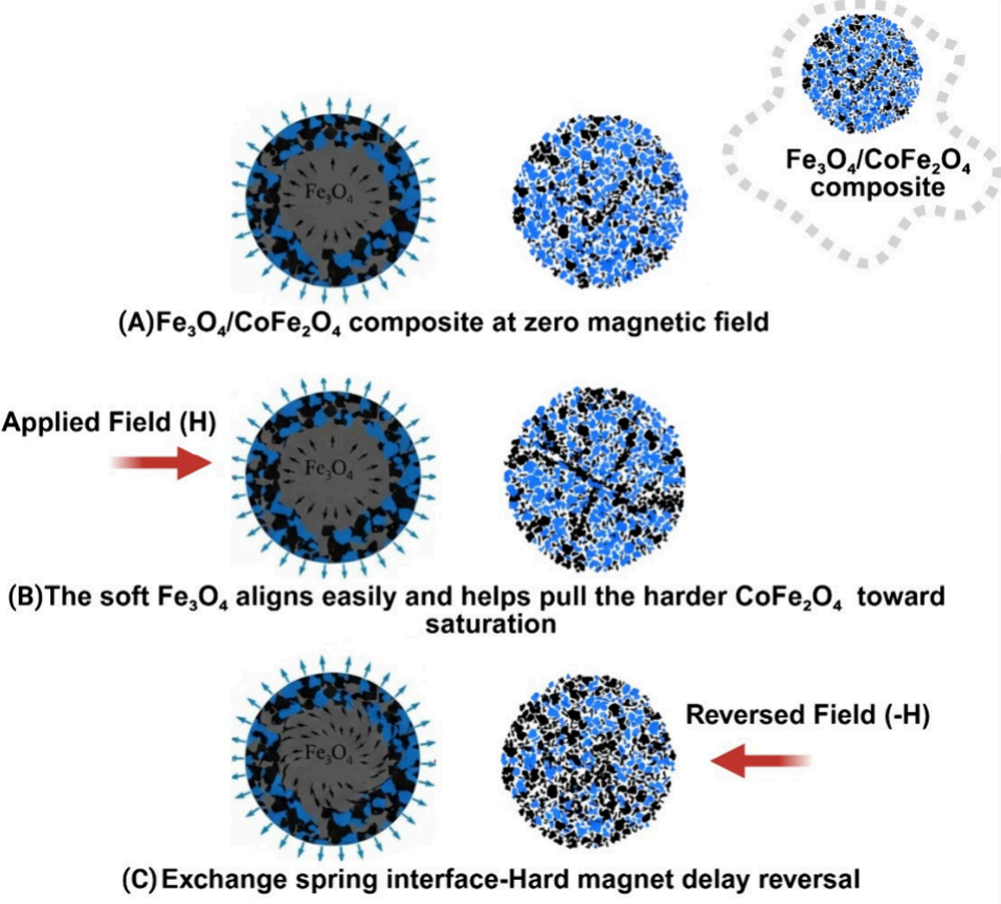
Schematic illustration of the exchange coupling in the Fe_3_O_4_/CoFe_2_O_4_ composite under varying magnetic fields. (A) At zero field (H = 0), Fe_3_O_4_ and CoFe_2_O_4_ phases exhibit a random arrangement. (B) Under applied field (+H), soft Fe_3_O_4_ aligns, facilitating alignment of hard CoFe_2_O_4_ toward saturation. (C) Field reversal (−H) induces delayed reversal of CoFe_2_O_4_ due to interfacial exchange coupling.

### 4.3 Pedagogical Goals

The primary pedagogical goal of this laboratory experiment is to engage students in the process of synthesis, characterization, and interpretation of functional nanomaterials through real-world experimental procedures. By integrating preparation techniques and characterization methods such as coprecipitation synthesis, X-ray diffraction, and vibrating sample magnetometry, students gain hands-on experience with methods commonly used in materials chemistry and nanotechnology research. Through analyzing XRD patterns, students are trained to identify crystalline phases, assess crystallite sizes, and recognize structural features. Similarly, interpreting VSM data introduces learners to the hysteresis loop and key magnetic parameters such as saturation magnetization, coercivity, and retentivity, and how they correlate with nanoparticle size, composition, and structural interfaces in the context of magnetic exchange coupling. This experience fosters the development of essential research skills, including critical analysis, data interpretation, and the ability to correlate experimental results with structural and functional properties of nanomaterials.

## 5 Conclusions

These laboratory experiments provide students with direct experience in synthesizing magnetite-based composites, offering authentic engagement with advanced materials chemistry and engineering. Through coupling Fe_3_O_4_ with TiO_2_ and CoFe_2_O_4_, students explore the concept of magnetic exchange coupling and examine how different partners modify the magnetic response and potential functional applications of the composites.

Students also learn to process and interpret data from two key characterization techniques, XRD and VSM, including plotting, analyzing, and critically discussing their results. Group discussion of the data is emphasized to foster scientific dialogue and develop critical thinking in quantitative analysis. Overall, the integration of synthesis, structural characterization, and magnetic property evaluation provides a robust, inquiry driven learning experience aligned with current research practice; this approach has already been successfully implemented in two REU cohorts, with one student contributing as a coauthor to the present work.

## Supplementary Material





## Notes

Laboratory guide for students, providing basics concepts and practical instructions for sample preparation, outlining the experimental steps, and explaining how to interpret the results they obtain. Laboratory Guide REU/Magnetics-Program (PDF, DOCX)

## ‡

Alan Levin Department of Mechanical and Nuclear Engineering, Kansas State University, Manhattan, KS 66503, USA, cecastano@ksu.edu

## †

J.L. and K.H. are undergraduate students.
