# Supplementary material for: Exploring Magnetic Exchange Coupling: Synthesis and Characterization of Magnetite-Based Composites
Source: J Chem Educ. 2026 Feb 19;103(3):1620–8. doi: 10.1021/acs.jchemed.5c01804 (PMC12980721; doi:10.1021/acs.jchemed.5c01804)
Supplement: Supplementary file 1 [file ed5c01804_si_001.pdf]

VCU College of Engineering

**Virginia Commonwealth University**

**College of Engineering**

**Mechanical and Nuclear Engineering Department**

# Laboratory Guide

## REU/Magnetics- Program

**Prepared by**

**Dr. Carlos E. Castano**

**Dr. Mostafa Gamal**

**August 2025**

| Lab Guide Index | |
|---|---|
| **Section** | **Page Number** |
| **Preface** | 3 |
| **Laboratory Safety Rules** | 4-5 |
| **Laboratory Report Guidelines and Example** | 6-7 |
| **Experiment 1: Synthesis of Magnetite ($Fe_3O_4$) Nanoparticles** | 8-14 |
| **Experiment 2: Preparation of Magnetite-Based Composites and Magnetic Exchange Coupling Concept** | 15-22 |
| **Experiment 3: X-ray Diffraction (XRD)** | 23-37 |
| **Experiment 4: Measurement of Magnetic Properties by Vibrating Sample Magnetometry (VSM)** | 38-45 |

# PREFACE

This laboratory guide has been prepared as a companion and guide for students participating in the Research Experiences for Undergraduates (REU) program in magnetics. The experiments and activities included here are designed to provide students with hands-on experience at the intersections of chemistry, materials science, physics, and engineering. By working directly with magnetic materials and their properties, students will gain a deeper appreciation of both the scientific principles and the practical applications underlying this rapidly advancing field.

The guide introduces a range of experimental techniques, from the synthesis and processing of materials to their structural, chemical, and magnetic characterization. Students are encouraged to move beyond following instructions toward developing an experimental mindset, asking questions, designing variations, analyzing results, and thinking critically about sources of error and interpretation. In doing so, they will not simply carry out experiments, but engage the process of research itself: discovery, iteration, and reflection.

The main goal is to provide a framework that fosters technical skills, problem-solving abilities, and scientific communication, while also conveying the excitement of collaborative research. We hope these experiments will serve both as training in core methods of magnetics and as inspiration for each student's future contributions to science and engineering.

## Laboratory Safety Rules

Material science laboratories are generally safe environments to work in, as long as all safety protocols are strictly followed. Each student needs to familiarize themselves with these regulations before commencing any laboratory activities. Adherence to these safety rules is crucial not only for your own protection but also for the safety of your peers.

### General Rules of Safety

1. Identify the location and learn how to use the fire extinguisher and the emergency shower and eyewash in the lab.

2. Become familiar with all exits (special emergency escape) from the lab.

3. Leave the lab immediately and quietly when you hear the building alarm.

4. Never perform any unauthorized experiments.

5. No experimental work can be done unless the instructor or the graduate students/postdoc are present.

6. Lab coat and safety goggles must be always worn while in the lab. The lab coat should be buttoned.

7. If you have long hair, you should tie it back so that it cannot catch fire or fall into your work.

8. Shorts or skirts cut above the knee are not allowed in the lab.

9. Open-toe shoes, sandals, or high-heel shoes are not allowed in the lab. You must wear shoes that completely cover your feet.

10. Do not eat, drink, or smoke in the lab at any time.

11. Never drink water from the lab faucet.

12. Consider all chemicals in the lab as hazardous.

13. Never taste any solid or liquid chemical.

14. Never smell directly any vapors from the test tube.

15. Do not touch your hands to your mouth or face during the experiment.

16. You must perform any experiment producing unpleasant odors or gases in the fume hood.

17. Never pick up hot objects with your bare hands.

18. Never point at a test tube that you are heating at your neighbor or yourself.

19. If you break glassware, clean up all broken glassware immediately. Never pick up broken glass with your bare hands.

20. Do not use flammable organic compounds and solvents such as alcohols, acetone, and ether near open flame.

21. If chemicals came in contact with your skin or eyes, wash immediately with plenty of running water for at least 10 minutes using an eyewash and a safety shower. (ASK FOR HELP).

As part of their research experience, students must submit a report summarizing the experiments conducted. This requirement is vital for enhancing their skills in effective reporting and presenting findings. Furthermore, each experiment concludes with a series of questions designed to assess students' comprehension of the material.

# Laboratory Report Example

**Experiment Number/Title:** ___________________________
**Date of Experiment:** ___________________________
**Submission Date:** ___________________________
**Name:** ___________________________
**Lab Partner(s):** ___________________________

**1. Title of the Experiment**

Provide a concise, specific title that clearly indicates the focus of the experiment.

---

**2. Aim/Objectives of the Experiment**

State the key aim or objective(s) of the experiment in 1-3 sentences. Indicate which concept, process, or principle is being investigated.

**3. Experimental Methods/Procedure**

- Summarize the experimental procedure in your own words.
- Include the name of equipment, materials, and techniques used.
- Note any deviations or modifications made from the standard procedure.

**4. Observations and Results**

- Present your experimental data clearly using **tables, graphs, and figures** as appropriate.
- Ensure all figures/tables are numbered, titled, and labeled with units.
- Include both qualitative observations (e.g., color change, precipitate formation) and quantitative data (measurements, values, calculated results).
- Highlight noticeable trends, expected vs. actual findings, and any anomalies.

**5. Discussion & Response to Questions**

- Answer the assigned post-lab questions in complete sentences, supported by your data and theory.
- Provide analysis of your results and explain how they relate to the experimental objective.
- Discuss sources of error and suggest improvements to the experimental design.
- Relate findings to underlying chemical engineering concepts or industrial applications.

**6. References (if applicable)**

List any sources you referred to. Use a consistent referencing style (e.g., APA, IEEE, or journal-specific format).

**General Notes for Writing**

- Reports must be written **individually** in your own words, even if data were shared.
- Use **scientific and professional language**. Avoid casual tone.
- Late submissions will **not** be accepted.
- Numerical values must include units and, where applicable, uncertainty/error estimates.
- Keep the report concise (typically 4–7 pages, excluding raw data appendices, unless otherwise specified).

# Experiment 1: Synthesis of Magnetite ($Fe_3O_4$) Nanoparticles via Co-precipitation

## Aim

This experiment aims to synthesize uniform magnetite ($Fe_3O_4$) nanoparticles using the co-precipitation method and understand the chemical and physical principles involved.

## Background

Magnetism is a fundamental physical phenomenon associated with the motion of electric charges, leading to the creation of magnetic fields. At its core, magnetism arises from two primary sources: the orbital motion of electrons around atomic nuclei and the intrinsic spin of electrons. These effects combine to produce magnetic moments in materials, which can interact with external magnetic fields. Magnetism plays a crucial role in various natural processes and technological applications, ranging from navigation and data storage to medical imaging and materials science.

*There are several distinct types of magnetism, each characterized by the behavior of materials in response to external magnetic fields:*

1. **Diamagnetism**: This is a weak form of magnetism exhibited by all materials, where they create an opposing magnetic field when subjected to an external magnetic field. Diamagnetic materials have no net magnetic moment in the absence of an external field, and their magnetic susceptibility is negative. Common examples include bismuth, copper, and graphite.
2. **Paramagnetism**: Paramagnetic materials have unpaired electrons, resulting in a net magnetic moment. When exposed to an external magnetic field, these materials become weakly magnetized in the direction of the field. However, this magnetization disappears once the external field is removed. Examples of paramagnetic materials include aluminum, platinum, and certain metal ions.
3. **Ferromagnetism**: This is a strong form of magnetism found in materials like iron, cobalt, and nickel. Ferromagnetic materials have regions called magnetic domains, where the magnetic moments of atoms are aligned in the same direction. When an external magnetic field is applied, these domains can grow, leading to a significant net magnetization. Unlike paramagnetism, ferromagnetism allows materials to retain their magnetization even after the external field is removed, resulting in permanent magnets.
4. **Ferrimagnetism**: Similar to ferromagnetism, ferrimagnetism occurs in materials with opposing magnetic moments that are unequal. This results in a net magnetization. Ferrimagnetic materials, such as magnetite ($Fe_3O4$), often consist of two different types of ions with different magnetic moments, leading to complex magnetic behavior.
5. **Antiferromagnetism**: In antiferromagnetic materials, adjacent magnetic moments align in opposite directions, canceling each other out. This results in no net magnetization in the absence of an external magnetic field. Antiferromagnetic behavior is common in transition metal oxides, such as manganese oxide.
6. **Superparamagnetism**: This phenomenon occurs in nanoscale magnetic particles, where thermal fluctuations can overcome magnetic interactions. Superparamagnetic materials can be magnetized in the presence of an external magnetic field, but do not retain magnetization once the field is removed. This property is fundamental in applications such as biomedical imaging and drug delivery.

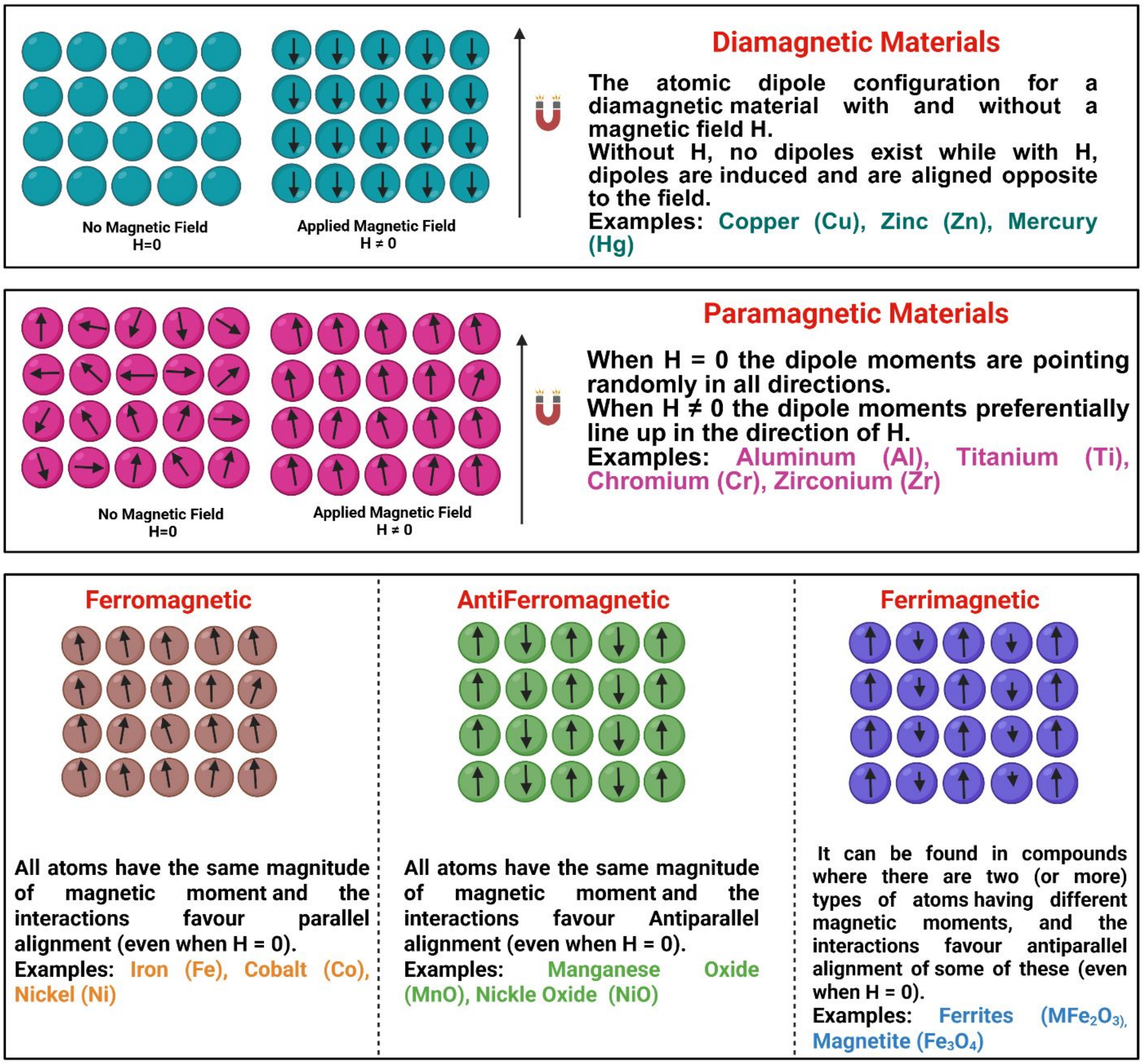


**Figure 1. Different types of magnetic materials.**

## Magnetic anisotropy

Magnetic anisotropy is an important term in understanding the magnetic properties of materials as it can be explained as that the magnetic moments in a material have preferred directions of alignment inside the crystal or particle. Because of this preference, the magnetization tends to stay along certain directions and resists being rotated away from them by an external field.

## Role in different magnetic materials

- **In ferromagnetic and ferrimagnetic materials,** this directional preference is strong, so once the magnetization is aligned along an easy direction, it tends to remain there even after the field is removed. This resistance to rotation gives rise to coercivity and remanent magnetization, which are characteristic of "harder" magnetic behavior.
- **In paramagnetic,** the anisotropy is weak and there is no stable spontaneous magnetization, so they do not show hysteresis loops or remanence; their magnetization simply follows the applied field and disappears when the field is removed.

- **In superparamagnetic (nanoparticles),** each particle is intrinsically ferromagnetic or ferrimagnetic, but the particles are so small that the anisotropy energy barrier becomes comparable to the thermal energy. As a result, the magnetization direction can flip randomly over time, averaging to zero and giving negligible coercivity on experimental timescales, even though the particles are made of a "magnetic" material.

Magnetite ($Fe_3O_4$) is a naturally occurring iron oxide that possesses a unique crystal structure known as inverse spinel. This structure is characterized by the arrangement of iron ions, where $Fe^{+3}$ ions occupy tetrahedral sites, while both $Fe^{+3}$ and $Fe^{+2}$ ions occupy octahedral sites. The distinctive arrangement of these ions leads to remarkable electronic and magnetic properties that make magnetite a subject of extensive research across materials science and engineering disciplines.

One of the most significant features of magnetite is its strong ferrimagnetism. In ferrimagnetic materials, the magnetic moments of different ions are aligned in opposite directions, but due to differing magnitudes, a net magnetization is achieved. In magnetite, the $Fe^{+3}$ ions, with their higher oxidation state, contribute a greater magnetic moment than the $Fe^{+2}$ ions, resulting in a collective magnetic behavior that is both robust and versatile. The magnetic properties are further enhanced by the presence of oxygen anions, which facilitate exchange interactions between the iron ions, influencing the overall magnetic ordering.

Generally, particle size critically influences the magnetic properties of materials, particularly magnetic nano-materials, by dictating the transition between multi-domain, single-domain, and superparamagnetic states (Figure 2). As particle size decreases below the critical single-domain threshold often around 20-80 nm depending on the material like magnetite or ferrites coercivity (Coercivity is the strength of the reverse magnetic field needed to drive the magnetization of a previously saturated material back to zero) initially rises due to enhanced magnetic anisotropy and uniform spin alignment, but then drops sharply in the superparamagnetic regime where thermal energy overcomes anisotropy barriers, leading to zero remanence and coercivity.

Smaller particles also exhibit reduced saturation magnetization (the maximum magnetization a magnetic material can reach when all of its magnetic moments are fully aligned with an applied field and no further increase in the field produces a significant increase in magnetization) from surface effects, such as spin disorder or dead layers, and cation redistribution, which narrow electronic bandwidths and lower Curie temperature (it is the critical temperature above which ferromagnetic or ferrimagnetic materials lose their permanent magnetism and transition to a paramagnetic state). This size dependence is vital for tailoring nanomaterials in applications like magnetic nanofluids, biomedical imaging, and data storage.

When magnetite particles are reduced to the nanoscale, they exhibit super-paramagnetism characteristics. In this state, the nanoparticles can be easily magnetized in the presence of an external magnetic field, but they do not retain any residual magnetization once the field is removed. This property is particularly advantageous for various applications, including biomedical imaging, magnetic separation, and targeted drug delivery, as it prevents particle aggregation while allowing

for strong magnetic responsiveness. Superparamagnetic behavior ensures that nanoparticles remain stable and functional in biological environments, which is crucial for their utility in many fields especially medical applications.

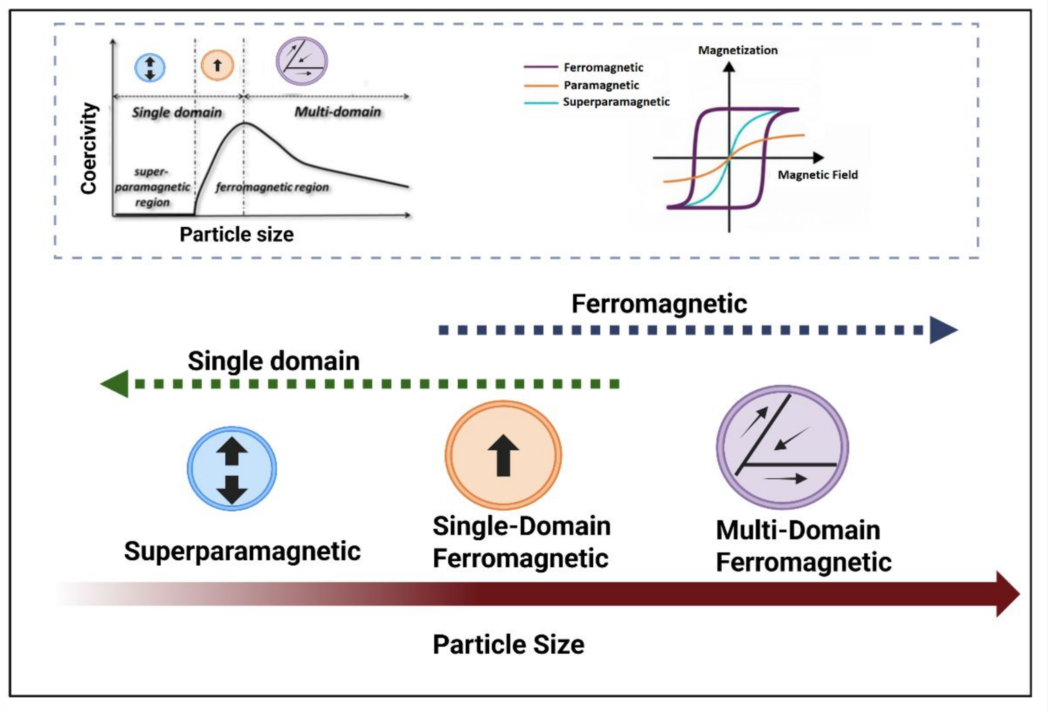


**Figure 2. Schematic illustration of the dependence of coercivity and magnetic behavior on particle size.**

**Synthesis of magnetite ($Fe_3O_4$) by co-precipitation method**

The co-precipitation synthesis method is one of the most widely adopted techniques for producing magnetite nanoparticles due to its simplicity, cost-effectiveness, and ability to yield high-purity products. This method is based on forming solid particles by rapidly converting dissolved metal ions into an insoluble phase through using precipitating agent (such as NaOH, $NH_4OH$, or carbonate) which controls when and how the solid phase forms. It raises the pH and changes the solution chemistry so that dissolved metal ions become supersaturated and convert into insoluble hydroxide or oxide nuclei.

In the preparation of the magnetite, co-precipitation involves the controlled precipitation of $Fe^{+2}$ and $Fe^{+3}$ ions from aqueous solutions under alkaline conditions. To achieve stoichiometric $Fe_3O_4$, a 2:1 molar ratio of $Fe^{3+}$ to $Fe^{2+}$ is maintained throughout the synthesis process. The reaction can be represented as follows:

$$Fe^{3+} + Fe^{2+} + 8OH^{-} \rightarrow Fe_3O_4 + 4H_2O$$

Several parameters significantly influence the resulting characteristics of the magnetite nanoparticles. Temperature plays a crucial role; a reaction temperature of around 70°C enhances the nucleation and growth rates, leading to well-defined nanoparticles. The pH of the solution is also critical, typically maintained between 10 and 11 to promote the precipitation of iron hydroxides, which subsequently convert into magnetite. Additionally, the stirring rate affects the uniformity of particle size and shape by ensuring a homogenous distribution of reactants during the synthesis process.

By carefully controlling these parameters, researchers can tailor the size, shape, and crystallinity of magnetite nanoparticles, which are essential for optimizing their performance in various applications. Understanding the synthesis, properties, and behavior of magnetite at both macroscopic and nanoscopic scales is vital for advancing its applications in modern science and technology. The unique combination of magnetic properties and the ability to manipulate particle characteristics makes magnetite a promising material for future innovations in fields ranging from biomedicine to environmental science.

**Experimental Procedure**

**Materials:**

- Iron (III) chloride hexahydrate ($FeCl_3 \cdot 6H_2O$)
- Iron (II) sulfate heptahydrate ($FeSO_4 \cdot 7H_2O$)
- Sodium hydroxide (NaOH, 2 mol $L^{-1}$ solution)
- Deionized water

**Apparatus:**

- 250 mL beaker
- Hot plate with magnetic stirrer
- Thermometer
- pH meter or pH strips
- Centrifuge
- Vacuum oven
- Strong magnet

**Steps (Figure3)**:

1. Dissolve 5.40 g of ferric chloride hexahydrate ($FeCl_3 \cdot 6H_2O$) in deionized water using a 100 mL volumetric flask to prepare a 0.2 mol $L^{-1}$ $FeCl_3 \cdot 6H_2O$ solution. Similarly, dissolve 2.78 g of ferrous sulfate ($FeSO_4.7H_2O$) in 100 mL volumetric flask to prepare a 0.1 mol $L^{-1}$

FeSO$_4$ solution. Mix each solution thoroughly until completely dissolved to ensure homogeneity.

2. Heat the solution to 60°C with constant stirring for 15 min.
3. Add 2 mol L$^{-1}$ NaOH solution (4 g of NaOH in 50 mL water) dropwise until pH reaches 10-11, observing formation of black $Fe_3O_4$ precipitate.
4. Continue stirring at the same temperature for 30 minutes for particle growth.
5. The solution was first allowed to cool, then transferred into centrifuge tubes and centrifuged at 5000 rpm for 10 min. The supernatant was carefully decanted to separate the nanoparticle pellet.
6. Wash the precipitate twice with deionized water to remove residual ions: After decantation, add approximately 20 mL of deionized water to the precipitate, gently swirl or vortex the mixture for 1-2 minutes to resuspend the particles, re-centrifuge, then carefully decant the supernatant. Repeat this washing step once more with a fresh 20 mL portion of deionized water using the same procedure to ensure thorough removal of unreacted salts and impurities.
7. Dry under vacuum at 40°C overnight.

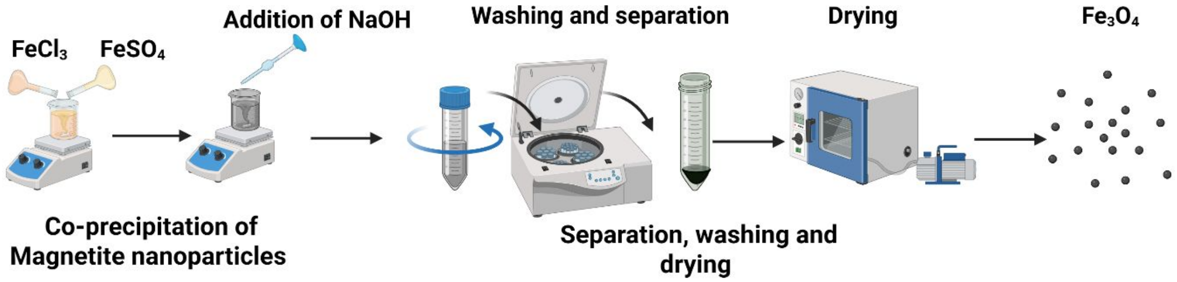


**Figure 3. schematic diagram for the preparation of the magnetite ($F_3O_4$).**

## Safety Notes

- Wear gloves, goggles, and lab coat at all times.
- Handle NaOH solutions and pellets with extreme care, as they are highly corrosive and can cause severe chemical burns to skin, eyes, and mucous membranes upon contact. In case of skin contact, immediately flush the affected area with copious amounts of running water for at least 15 minutes and seek medical attention. For eye exposure, irrigate with sterile water using an eyewash station for 15-20 minutes while holding eyelids open, then obtain urgent medical care. Never add water to NaOH; always add base to water slowly while stirring to control heat evolution and prevent splattering.
- Avoid inhalation of powders. Use a ventilated fume hood when dissolving powders such as NaOH pellets, metal salts (e.g., $FeCl_3$, $FeSO_4$), to minimize airborne particle dispersion.
- Dispose of waste following the institutional guidelines.

## Expected Results

Black magnetic magnetite nanoparticles formed that can be attracted by a magnet.

**References**


1. Mushnikov, N.V., 2022. Basics of magnetism and magnetic materials. In Magnetic Materials and Technologies for Medical Applications (pp. 23-55). Woodhead Publishing.
2. Krishnan, K.M., 2016. Fundamentals and applications of magnetic materials. Oxford University Press.
3. Petcharoen K, Sirivat AJ. Synthesis and characterization of magnetite nanoparticles via the chemical co-precipitation method. Materials Science and Engineering: B. 2012 Mar 25;177(5):421-7.
4. Wu, C. and Jin, J., 2022. Frontiers in magnetic materials: from principles to material design and practical applications. CRC Press.
5. Shen L, Qiao Y, Guo Y, Meng S, Yang G, Wu M, Zhao J. Facile co-precipitation synthesis of shape-controlled magnetite nanoparticles. Ceramics International. 2014 Jan 1;40(1):1519-24.
6. Aphesteguy, J.C., Kurlyandskaya, G.V., De Celis, J.P., Safronov, A.P. and Schegoleva, N.N., 2015. Magnetite nanoparticles prepared by co-precipitation method in different conditions. Materials Chemistry and Physics, 161, pp.243-249.
7. Losito, D.W., Souza, N.I., Martins, T.S., Britos, T.N., Schumacher, M.L. and Haddad, P.S., 2024. A review of superparamagnetic nanoparticles applications and regulatory aspects in medicine and environmental areas. *Journal of Materials Science*, *59*(34), pp.16038-16068.


**Post-Lab Questions**

1. Compare and contrast ferromagnetism and paramagnetism. What are the key differences in their behavior in the presence of an external magnetic field, and what are some examples of materials that exhibit each type?
2. What are magnetic domains, and how do they contribute to the overall magnetic properties of ferromagnetic materials? How does the alignment of these domains change when an external magnetic field is applied?
3. Why is it important to maintain a 2:1 molar ratio of $Fe^{3+}$ to $Fe^{2+}$ ions during the synthesis of magnetite? How does this ratio influence the properties of the resulting compound?
4. How does the pH of the solution affect the precipitation of magnetite? What is the optimal pH range for this reaction, and what are the consequences of deviating from this range?
5. What is superparamagnetism, and why is this property particularly beneficial for the use of magnetite nanoparticles in biomedical applications?
6. Identify two methods that could be used to characterize the synthesized magnetite nanoparticles. What specific properties would each method help to determine?

# Experiment 2: Preparation of Magnetite-Based Composites and Magnetic Exchange Coupling Concepts

## Aim

This experiment aims to synthesize magnetite-based composites by coupling $Fe_3O_4$ nanoparticles with titanium dioxide ($TiO_2$) and cobalt ferrite ($CoFe_2O_4$), and to understand the fundamental principles and effects of magnetic exchange coupling on the magnetic properties.

## Background

Magnetism is a fundamental force of nature arising from the motion and intrinsic spin of electrons within atoms. It is responsible for the ability of certain materials to attract or repel others and to generate magnetic fields. Magnetic characteristics describe how materials respond to applied magnetic fields and include key parameters such as magnetization, which is the magnetic moment per unit volume, indicating how strongly a material can become magnetized. Saturation magnetization ($M_s$) is the maximum magnetization when all magnetic moments align in the presence of an external field. Coercivity ($H_c$) measures the resistance of a material to becoming demagnetized, defined as the external magnetic field required to reduce magnetization to zero. Remanent magnetization ($M_r$) is the residual magnetization remaining after removing the external field. Magnetic materials are broadly categorized into soft and hard magnets based on their magnetic response and coercivity. Soft magnetic materials are easily magnetized and demagnetized, exhibiting low coercivity, high permeability, and minimal energy loss during magnetic cycling. These properties make soft magnets ideal for applications like transformer cores and electromagnetic devices, where the magnetic field changes frequently. In contrast, hard magnetic materials exhibit high coercivity, meaning they retain their magnetization even after the external magnetic field is removed, making them suitable as permanent magnets. Hard magnets show wide hysteresis loops with significant energy loss but provide stable, long-lasting magnetic fields essential for data storage, motors, and magnetic sensors. Table 1 illustrates the main differences between the hard and soft magnets.

Table (1): comparison between soft and hard magnets

| | Hard Magnetic Materials | Soft Magnetic Materials |
|---|---|---|
| **Magnetization** | Hard magnetic materials cannot be easily magnetized; they require strong external magnetic fields to align their magnetic domains. | Soft magnetic materials can be magnetized easily with relatively weak external magnetic fields due to mobile domain walls. |
| **Manufacturing Process** | Typically produced by rapid cooling (quenching) from high temperatures, resulting in a microstructure that stabilizes magnetic domains. | Usually produced by slow cooling and annealing processes that promote large, well-ordered grains for easy magnetization. |
| **Domain Behavior** | Domain walls in hard magnets are strongly pinned by defects and microstructural features, resisting movement and requiring high coercive fields ($H_c$). | Domain walls in soft magnets move freely with low resistance, allowing magnetization with minimal applied coercive field. |
| **Magnetic properties** | High retentivity (remanence) and high coercivity, meaning the material retains strong magnetization after removal of external fields. | Low retentivity and low coercivity, enabling the material to be easily demagnetized. |
| **Examples** | Alnico alloys (Al–Ni–Co), chromium steel, tungsten steel, carbon steel, and rare earth-based permanent magnets (e.g., NdFeB). | Iron-silicon (Fe-Si) alloys, ferrous-nickel alloys (Permalloy), ferrites, and garnets. |
| **Potential applications** | Used as permanent magnets in motors, generators, loudspeakers, magnetic recording media, and DC devices where stable magnetization is required. | Utilized in transformer cores, inductors, electromagnets, magnetic shielding, and any device requiring low hysteresis loss. |

***Magnetite and Magnetic Exchange Coupling***

Magnetite ($Fe_3O_4$) nanoparticles exhibit soft magnetic behavior, characterized by low coercivity and relatively high saturation magnetization. By creating composites with other materials like titanium dioxide and cobalt ferrite, it is possible to tailor the magnetic properties significantly through the phenomenon known as magnetic exchange coupling.

Magnetic exchange coupling is the way spins in one region of a material "feel" and influence the spins in a neighboring region, so that the two phases no longer behave independently under a magnetic field (see Figure 1). When a soft magnetic phase like $Fe_3O_4$ is coupled to a hard phase such as $CoFe_2O_4$, the spins at the interface lock together, and the hard phase partially controls how easily the soft phase can reverse its magnetization. As a result, macroscopic properties like coercivity, remanent magnetization, and even the effective saturation magnetization are shifted compared with either phase alone.

**Examples on magnetic exchange coupling**

**Soft-hard exchange coupling ($Fe_3O_4$/$CoFe_2O_4$)**

Cobalt ferrite ($CoFe_2O_4$) is a prominent hard magnetic material characterized by high coercivity and moderate saturation magnetization. When combined with magnetite ($Fe_3O_4$), the two magnetic phases exchange couple at their interface, resulting in composite magnetic behavior that balances the high coercivity of $CoFe_2O_4$ with the high saturation magnetization of $Fe_3O_4$. This coupling enhances magnetic anisotropy and thermal stability, opening potential applications in permanent magnets and high-density magnetic storage.

In a well-coupled nanocomposite, the soft $Fe_3O_4$ has low anisotropy and wants to reverse easily, while $CoFe_2O_4$ has high anisotropy and resists reversal.

- Exchange at the interface forces the spins in the soft phase to rotate more coherently with those in the hard phase, which:
- Increases effective anisotropy and coercivity compared with pure $Fe_3O_4$.
- Maintains or even boosts remanence, because many $Fe_3O_4$ spins remain aligned with the hard phase after the external field is removed.

Conceptually, the hard phase acts like an internal "*template*" that stabilizes the direction of magnetization, while the soft phase contributes high magnetization and reduces brittleness or density. The key requirement is nanoscale mixing with interfaces thinner than or comparable to the soft phase's exchange length so that the whole composite behaves like a single, exchange-spring system rather than two decoupled materials.

**Soft-non-magnetic coupling ($Fe_3O_4$/$TiO_2$)**

Titanium dioxide ($TiO_2$) is a non-magnetic semiconductor extensively used in photocatalysis and environmental applications. The process of coupling $TiO_2$ with $Fe_3O_4$ can provide the magnetite nanoparticles surface passivation, chemical stability, and enhanced dispersibility without imparting any magnetic properties. This composite structure not only preserves the soft magnetic characteristics of magnetite but also protects the particles from oxidation and agglomeration. Furthermore, the presence of $TiO_2$ enhances photocatalytic applications by combining the magnetic separability of $Fe_3O_4$ with the photocatalytic capabilities of $TiO_2$.

When $Fe_3O_4$ is embedded in a non-magnetic matrix such as $TiO_2$, there is still an interface, but $TiO_2$ does not carry long-range magnetic order. There is no hard phase to pin the $Fe_3O_4$ spins, so classical soft–hard exchange-spring behavior is absent.

*However, the $TiO_2$ matrix can still modify $Fe_3O_4$'s magnetic properties indirectly by:*

- Controlling particle size and spacing isolating $Fe_3O_4$ nanoparticles reduces interparticle dipolar interactions and can decrease coercivity and remanence, often moving the system toward superparamagnetic or "ultra-soft" behavior if the particles are small enough.
- Changing surface chemistry and strain: bonding at the $Fe_3O_4/TiO_2$ interface, lattice mismatch, or interdiffusion can alter local anisotropy, spin canting, and the fraction of magnetically "dead" surface layers, which affects both $M_S$ and coercivity.
- Modifying thermal and structural stability: $TiO_2$ can prevent aggregation and phase transformation of $Fe_3O_4$, keeping the soft phase nanosized and stabilizing a particular magnetic state.

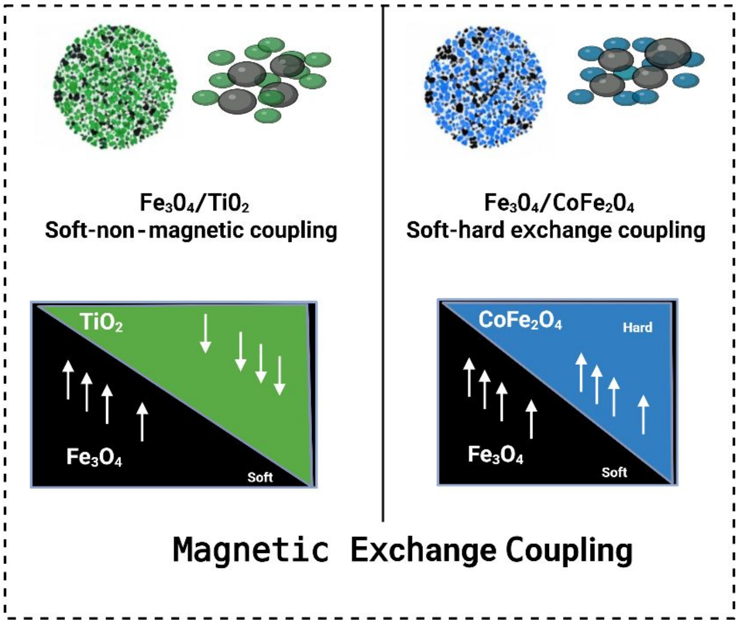


**Figure 1. Schematic illustration of soft-non-magnetic coupling in $Fe_3O_4/TiO_2$, and soft-hard exchange coupling in $Fe_3O_4/CoFe_2O_4$.**

## Preparation of different composites based on magnetite nanoparticles

The $Fe_3O_4/CoFe_2O_4$ and $Fe_3O_4/TiO_2$ composites were synthesized by a co-precipitation route analogous to that used for the bare magnetite nanoparticles, using pre-formed $Fe_3O_4$ as a magnetic substrate onto which the secondary phases were grown. This approach ensures that the new phase forms directly on the magnetite surface, favoring well-integrated composite structures rather than simple physical mixtures.

## Role of pretreatment and surface chemistry

Prior to composite growth, the $Fe_3O_4$ particles were functionalized with citric acid, which acts as a multidentate ligand. The adsorbed citrate introduces negatively charged carboxylate groups on the surface, improving colloidal stability by electrostatic repulsion and thus suppressing

aggregation. At the same time, these –COOH/–COO⁻ groups provide chemically active sites for heterogeneous nucleation of $TiO_2$ or $CoFe_2O_4$ during co-precipitation, promoting the formation of a well-distributed secondary phase on the magnetite. This surface modification enhances interfacial bonding between $Fe_3O_4$ and the grown oxide or ferrite, which is critical for achieving uniform microstructures, high nucleation density, and strong interfacial magnetic exchange coupling in the final composites.

**Experimental Procedure**

**Materials:**

- $Fe_3O_4$ nanoparticles synthesized in Experiment 1
- Citric acid (3 mol $L^{-1}$ solution)
- Titanium tetrachloride solution ($TiCl_4$)
- Cobalt nitrate hexahydrate ($Co(NO_3)_2 \cdot 6H_2O$)
- Iron(III) chloride hexahydrate ($FeCl_3 \cdot 6H_2O$)
- Sodium hydroxide (NaOH, 2 mol $L^{-1}$)
- HCl solution (1 mol $L^{-1}$)
- Deionized water

**Apparatus:**

- Hot plate with magnetic stirrer
- Thermometer
- pH meter or pH strips
- Vacuum oven
- Ultrasonic bath (for sonication).
- Centrifuge for separation.
- Dry glass syringe.

**Preparation procedures**

**Step 1: Pretreatment of $Fe_3O_4$ Nanoparticles**

1. Disperse 1 g of $Fe_3O_4$ nanoparticles in 50 mL of 3 mol $L^{-1}$ citric acid solution.
2. Sonicate for 10 minutes to facilitate chemisorption of citric acid molecules onto particle surfaces.
3. Separate by centrifugation or magnet, discard supernatant, and resuspend in deionized water.

***Two student groups (A and B) can be formed, each responsible for preparing one of the composite systems.***

**Step 2A: Preparation of $Fe_3O_4/TiO_2$ Composite (Figure 2)**

1. In a fume hood, disperse the citric-acid-treated $Fe_3O_4$ nanoparticles in 50 mL deionized water with vigorous stirring.

2. Add 1.0 mL $TiCl_4$ dropwise using a dry glass syringe to 20 mL ice-cold (0-5°C) 1 mol $L^{-1}$ HCl with vigorous magnetic stirring. Continue stirring until a clear pale yellow $TiOCl_2$ solution forms (~10 min), maintaining temperature below 10°C to control exothermic HCl evolution.
3. Slowly add the pre-hydrolyzed $TiOCl_2$ solution to the stirred $Fe_3O_4$ suspension at room temperature using dry glass pipette. Heat the solution to 80°C, then add 2 mol $L^{-1}$ NaOH solution dropwise to reach pH 10-12. Stir at 80°C for 30 min to complete hydrolysis. Cool to room temperature, separate the composite particles by centrifugation and decantation, and wash two times with deionized water to remove residual ions.
4. Dry the washed composite at 40 °C (under vacuum) overnight, then gently grind and store the $Fe_3O_4/TiO_2$ powder in a labeled container.

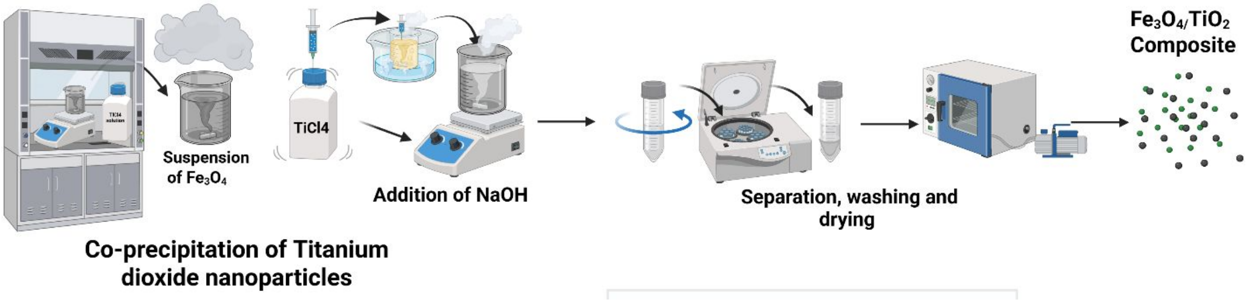


**Figure 2. Preparation procedure of $Fe_3O_4/TiO_2$.**

### Step 2B: Preparation of $Fe_3O_4/CoFe_2O_4$ Composite (Figure 3)

1. Dissolve $Co(NO_3)_2 \cdot 6H_2O$ and $FeCl_3 \cdot 6H_2O$ in deionized water (total volume 50 mL) using a Co:Fe molar ratio of 1:2 to obtain the precursor solution.
2. Add this precursor solution to the citric-acid-treated $Fe_3O_4$ suspension under vigorous stirring.
3. Add 2 mol $L^{-1}$ NaOH solution dropwise until the pH reaches ~10, then heat the mixture to about 70 °C and maintain stirring for 30 min to co-precipitate $CoFe_2O_4$.
4. Separate the resulting $Fe_3O_4/CoFe_2O_4$ composite by centrifugation and wash several times with deionized water to remove residual ions.

5. Dry the washed composite powder at 40 °C under vacuum, then gently grind and store in a labeled container.

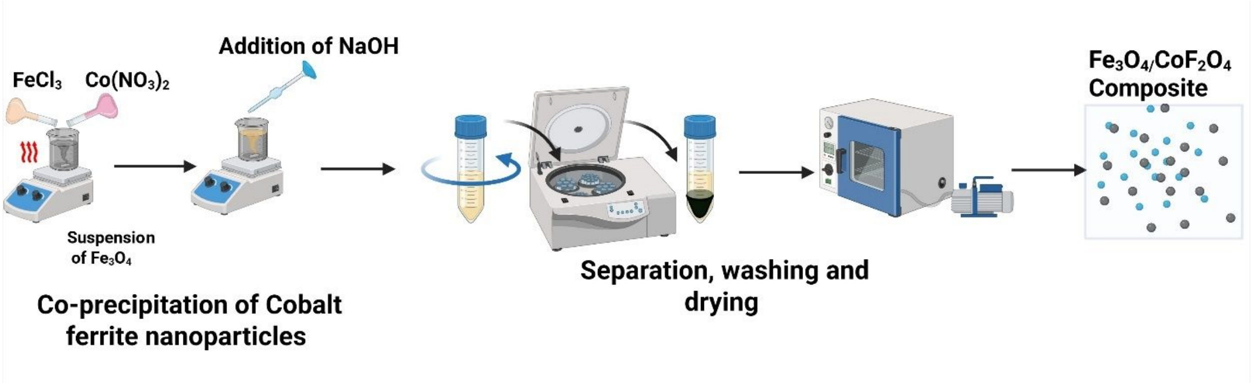


**Figure 3. Preparation procedure of $Fe_3O_4/CoFe_2O_4$.**

## Safety Notes

- $TiCl_4$ specific precautions

$TiCl_4$ is highly moisture-sensitive and fuming; even brief contact with atmospheric humidity generates dense white fumes of HCl and $TiO_2$ and can rapidly corrode equipment and irritate the respiratory tract. For this reason, all manipulations must be carried out inside a functioning chemical fume hood, with the sash kept as low as practicable, and only by researchers and students wearing full PPE (lab coat, chemical-resistant gloves, splash goggles, and preferably a face shield). The reagent bottle should remain tightly closed except during transfer, and all glassware used with $TiCl_4$ must be completely dry. Transfer is best performed with a dry glass syringe. Any spills or residues must be immediately contained with absorbent and neutralized with water.

- Cobalt salts should be handled with full PPEs including gloves, goggles and lab coat inside the fume hood and disposed of properly due to toxicity.
- Maintain careful pH and temperature control to avoid uncontrolled precipitation or hazardous conditions.

## Expected Results

- The formation of $Fe_3O_4/TiO_2$ and $Fe_3O_4/CoFe_2O_4$ composites for further studies on their magnetic properties and the phenomenon of magnetic exchange coupling

## References

1. Natekar, N.A. and Venugopal, A., 2024. Basics of magnetic materials and magnetism. In Magnetic Nanoparticles in Nanomedicine (pp. 3-34). Woodhead Publishing.
2. Liu, F., Hou, Y. and Gao, S., 2014. Exchange-coupled nanocomposites: chemical synthesis, characterization and applications. *Chemical Society Reviews*, *43*(23), pp.8098-8113.

3. Dheyab MA, Aziz AA, Jameel MS, Noqta OA, Khaniabadi PM, Mehrdel B. Simple rapid stabilization method through citric acid modification for magnetite nanoparticles. Scientific reports. 2020 Jul 1;10(1):10793.
4. Lee DS, Liu TK. Preparation of TiO2 sol using TiCl4 as a precursor. Journal of Sol-Gel Science and Technology. 2002 Sep;25(2):121-36.
5. Zhang Y, Yang Z, Yin D, Liu Y, Fei C, Xiong R, Shi J, Yan G. Composition and magnetic properties of cobalt ferrite nano-particles prepared by the co-precipitation method. Journal of Magnetism and Magnetic Materials. 2010 Nov 1;322(21):3470-5.

**Post-Lab Questions**

1. Explain the concept of magnetic exchange coupling. How does this interaction between magnetic spins in adjacent atoms or phases influence the magnetic properties of composite materials such as $Fe_3O_4/CoFe_2O_4$?
2. Identify and describe the key parameters used to quantify magnetic exchange coupling. How do these parameters relate to the magnetic behavior that can be observed in your prepared composites?
3. Explain why the coercivity of a composite magnetic material might increase when hard magnetic materials are coupled with soft magnetic materials.
4. Propose other materials or methods that could be used to create magnetic composites with tailored magnetic properties. Explain your choices briefly.
5. Explain the role of citric acid pretreatment in the formation of magnetite-based composites. How does this step influence the magnetic coupling?

# Experiment 3: X-ray diffraction (XRD)

## Aim

The objective of this experiment is to explore a key method for characterizing inorganic materials and examining their chemical phases and crystal structures. Specifically, it aims to illustrate how Bragg's Law establishes a relationship between the wavelength of incident X-rays, the angle of diffraction, and the interplanar spacing within a crystal. Through observation, analysis, and evaluation, this method can be utilized to determine lattice parameters and identify distinct material phases within crystalline substances.

## Introduction

### Historical Background

The discovery of X-rays occurred largely by accident, yet their significance was recognized immediately. In November 1895, Wilhelm Conrad Röntgen was experimenting with an early cathode ray tube when he observed that the faint green glow it produced could pass directly through various objects. Curious, he placed different materials in the beam's path and was astonished to see the shadowy image of the bones in his own hand projected onto a nearby screen. Röntgen documented his findings in a scientific paper, referring to the mysterious radiation as "X" to denote the unknown, and was later awarded the very first Nobel Prize in Physics for this groundbreaking discovery.

News of Röntgen's work spread quickly, inspiring others to explore the phenomenon. Thomas Edison, for example, immediately began testing alternative filament materials and, within just a few months, created the first practical fluoroscope for medical imaging. Continued research over the following years gradually clarified the nature of this unusual radiation. Despite occasional suggestions to rename them "Röntgen rays," the term "X-rays" endured and remains in use today.

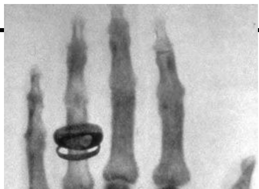

Wilhelm Röntgen

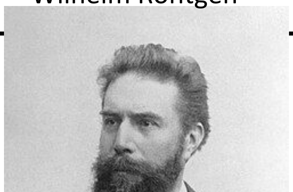

Following the discovery of X-rays, a major breakthrough came in 1912 when father-and-son physicists William Henry Bragg and William Lawrence Bragg demonstrated that X-rays could be used to probe the arrangement of atoms within crystals. They showed that when X-rays strike the regularly spaced planes of a crystal, the waves can reflect and interfere with each other in a predictable way. From this, William Lawrence Bragg derived a simple mathematical relationship now known as Bragg's Law which relates the wavelength of the radiation, the angle of reflection, and the distance between crystal planes. This discovery laid the foundation for the field of X-ray crystallography, providing scientists with a powerful method to determine the atomic and molecular structures of solid materials. For this pioneering work, the Braggs were jointly awarded the Nobel Prize in Physics in 1915, making William Lawrence Bragg, at age 25, the youngest-ever Nobel laureate in science.

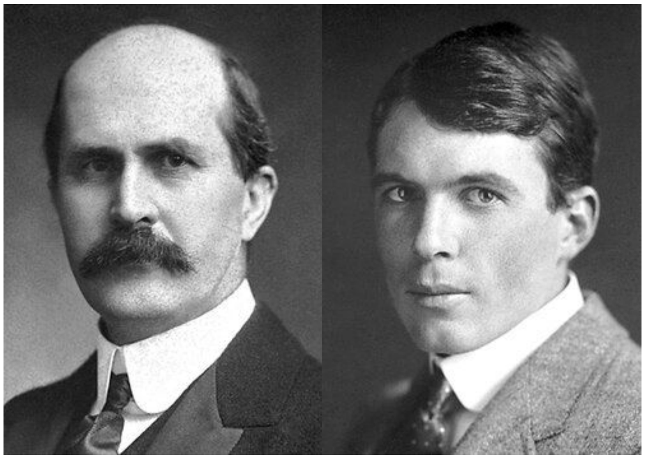

**William Henry Bragg** **William Lawrence Bragg**

*Photograph of William Lawrence Bragg published in 1916 in Sweden in Les Prix Nobel 1915, p. 103.*

*Photograph of William Henry Bragg published in 1916 in Sweden in Les Prix Nobel 1915, p. 101.*

*Public Domain Picture taken over 100 years ago.*

X-ray diffraction (XRD) is an essential technique in materials science for determining the crystal structure and lattice parameters of solids. Since each element and compound exhibits a unique crystalline arrangement and interatomic distances at room temperature, XRD provides a powerful "fingerprint" for material identification.

The main concept of XRD is based on that when an incident beam of X-rays strikes a crystalline sample, several interactions can occur, primarily absorption and scattering. Scattering phenomena may be:

- **Elastic scattering,** in which photons are deflected without energy loss, or
- **Inelastic scattering,** in which photons transfer part of their energy to the sample.

XRD is based on coherent elastic scattering, where the scattered waves maintain the same wavelength as the incident beam and can interfere with one another. Because X-rays have wavelengths on the same order of magnitude as interatomic spacings in crystals (~0.01–10 nm), periodic arrangements of atoms in solids act much like a three-dimensional diffraction grating for X-rays.

***The condition for constructive interference which produces observable diffracted peaks is described by Bragg's Law:***

$$n\lambda = 2d\sin\theta$$

**Where:**

**n is an integer (order of diffraction),**

**λ is the X-ray wavelength,**

**d is the interplanar spacing between crystallographic planes,**

**θ is the angle between the incident beam and the crystal planes.**

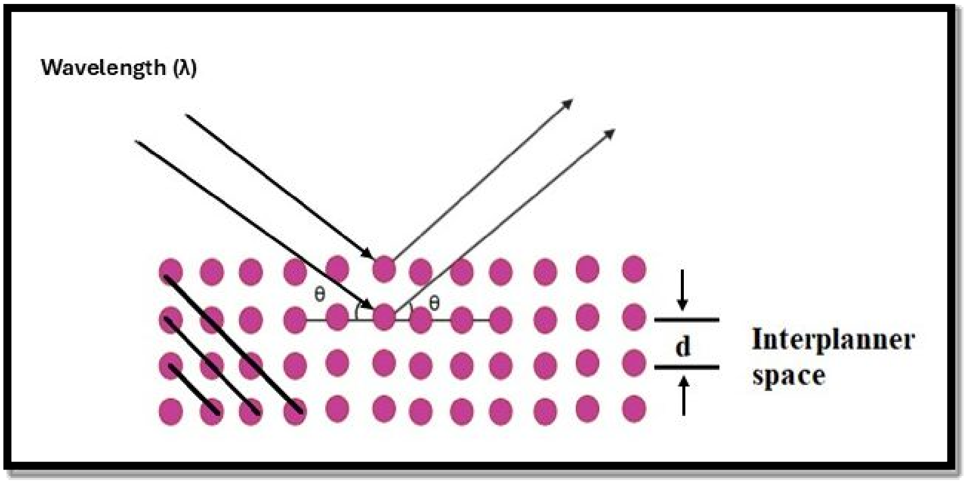


**Figure 2. Crystalline lattice showing the derivation of Bragg's law**

This relationship arises by considering the path difference between X-rays scattered by adjacent planes in the crystal lattice. When this path difference is equal to an integer multiple of the wavelength, constructive interference reinforces the diffracted signal, producing a measurable peak. Through measuring diffraction angles and knowing the wavelength of the X-rays, it is possible to calculate the interplanar spacing (d). From this, it can be deduced the unit cell dimensions, lattice type, and even identify unknown crystalline materials.

**Miller Indices (hkl)**

Miller indices are a standardized notation used to describe crystallographic planes and directions within a crystal lattice. They are essential in materials science, chemistry, and solid-state physics because they provide a concise way of identifying specific orientations in the periodic arrangement of atoms.

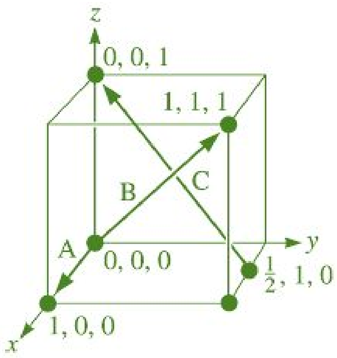


- **Crystal Points and Lattice**

A crystal lattice is an infinite, periodic arrangement of points (atoms or groups of atoms) in space. The lattice points form the underlying repetitive structure of a crystal. By connecting these points, one can define directions (lines between points) and planes (flat surfaces passing through points).

- **Miller Indices for Directions [XYZ]**

Miller indices can also be used to denote directions (vectors) within a crystal lattice. These are written as three integers enclosed in square brackets, for example, [XYZ] directions actually represent vectors between lattice points.

**How to determine direction indices:**

- **Determine** the vector components along the crystallographic axes in terms of unit cells a,b,c.
- **Expres**s these as the smallest set of integers X, Y, Z with no common factor.
- **Write** the indices in square brackets [XYZ].

While the concept can feel non-intuitive at first, it becomes clearer once you remember that Miller indices are essentially a way to translate geometric information (planes in a 3D grid) into a set of integers. These integers are directly tied to the relative intercepts of the plane with the unit cell axes.

**Steps to Determine Miller Indices for Planes**

**1. Identify the Plane**

Locate the crystal plane you wish to analyze within the unit cell.

**2. Determine Intercepts**

- Find where the plane intersects the axes of the unit cell (x, y, z).
- Record these intercepts as fractions of the unit cell dimensions.

**3. Take Reciprocals**

Take the reciprocal of each intercept. If an intercept is infinite (the plane is parallel to an axis), its reciprocal is zero.

**4. Clear Fractions**

If necessary, multiply the reciprocals by the least common multiple to eliminate any fractions.

**5. Assign Miller Indices**

The resulting integers are the Miller indices (h, k, l) for the plane.

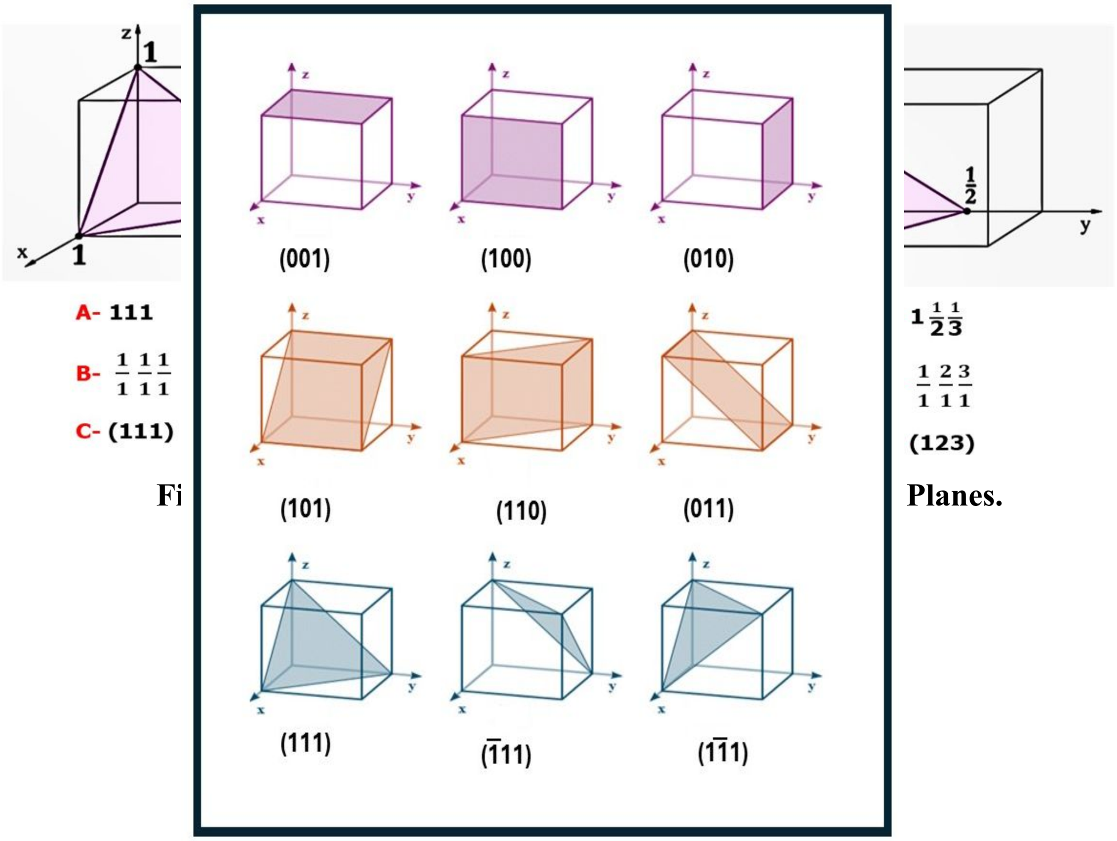


**Figure 3. Examples for different Miller indices planes.**

### X-ray diffraction types and operation

X-ray diffraction (XRD) is a fundamental characterization technique used to probe the crystal structure of materials across a wide range of length scales. In its most common form, powder X-ray diffraction (PXRD or XRPD) is applied to polycrystalline samples and enables phase identification, lattice parameter refinement, and estimation of crystallite size and microstrain. Single-crystal XRD (SCXRD) extends the method to high-quality single crystals, providing precise three-dimensional atomic structures, including space group and atomic coordinates. For thin films and surface-modified materials, grazing-incidence XRD (GIXRD) enhances surface sensitivity by using very low incidence angles, while high-resolution XRD (HRXRD) is tailored to epitaxial layers and multilayers, yielding detailed information on strain, lattice mismatch, and defect distributions. In these experiments, all diffraction measurements involving powdered samples will be carried out using powder X-ray diffraction (PXRD).

### Instrumentation

X-ray diffraction (XRD) instrumentation setup operates using 2θ scanning method in reflection mode, specifically employing the Bragg–Brentano geometry. In this configuration, the sample is fixed at the center of a goniometer, while both the X-ray source (tube) and the detector rotate synchronously on a circular path around the sample. The incident X-ray beam strikes the sample surface at an angle θ, and the detector simultaneously measures the diffracted X-rays at an angle of 2θ.

This coordinated motion ensures the angle between the incoming X-rays and the diffracted beam is always twice the incident angle (2θ), maintaining the conditions for symmetrical reflection. As the angles vary during scanning, certain values satisfy Bragg's Law, where constructive interference occurs, producing sharp diffraction peaks.

### XRD Instrumentation and Component Functions

**X-ray Tube (Source):** Generates the primary X-ray beam directed towards the sample. It typically contains a cathode that emits electrons, which strike a metal target to produce X-rays.

**Soller Slits:** Collimate the X-ray beam, reducing divergence and ensuring that the beam is well-directed and narrow, which improves resolution. There are two sets: one before the sample (primary optics) and one before the detector (secondary optics).

**Divergence Slit:** Controls the angular divergence of the incident beam on the sample, optimizing the beam size and intensity for better measurement accuracy.

**Anti-scatter Slit:** Placed near the sample to minimize the number of scattered X-rays reaching the detector, enhancing signal clarity and reducing background noise.

**Sample Holder/Goniometer Center:** Holds the sample fixed in place at the center of the circular motion. It allows precise alignment of the sample surface with respect to the incoming beam.

**Receiving Slit:** Positioned in front of the detector to further restrict the beam width and improve angular resolution by defining the exact diffracted beam accepted.

**Detector:** Measures the intensity of diffracted X-rays at varying angles (2θ). Its movement is synchronized with the X-ray source to maintain the 2:1 angular relation with the incident beam.

**Single Crystal Monochromator:** Sometimes placed in the secondary optics path to select a single wavelength from the polychromatic X-ray beam, improving measurement accuracy by filtering out unwanted wavelengths.

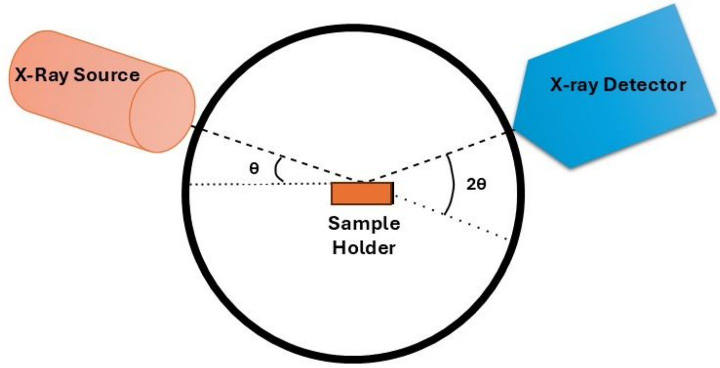

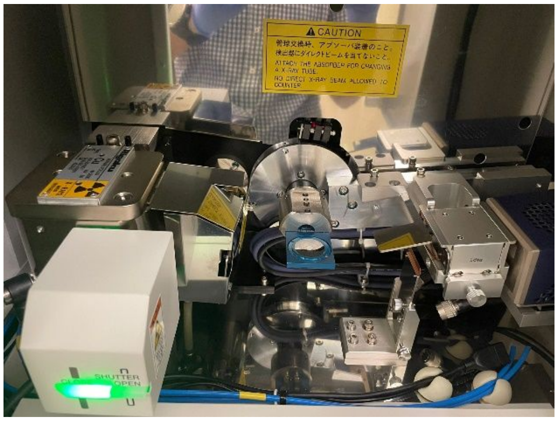


**Figure 4. XRD instrumentation diagram.**

## Sample Preparation

Proper sample preparation is critical to obtaining accurate and reliable X-ray diffraction data. First, there are different types of sample holders which can be used according to the material type.

### Types of sample holders

1. A stage adapter is provided to fit the zero-background silicon holders securely.
2. Aluminum wells without bottoms.
3. Glass wells with 0.2 mm depth can be replaced by custom holders made from aluminum or durable plastic.
4. Zero-backgtound silicon holders are used for general powder samples.
5. Silicon holders with multiple 0.2 mm wells are available for sample containment.
6. Silicon holders with/without wells and plastic caps are designed for air-sensitive samples.

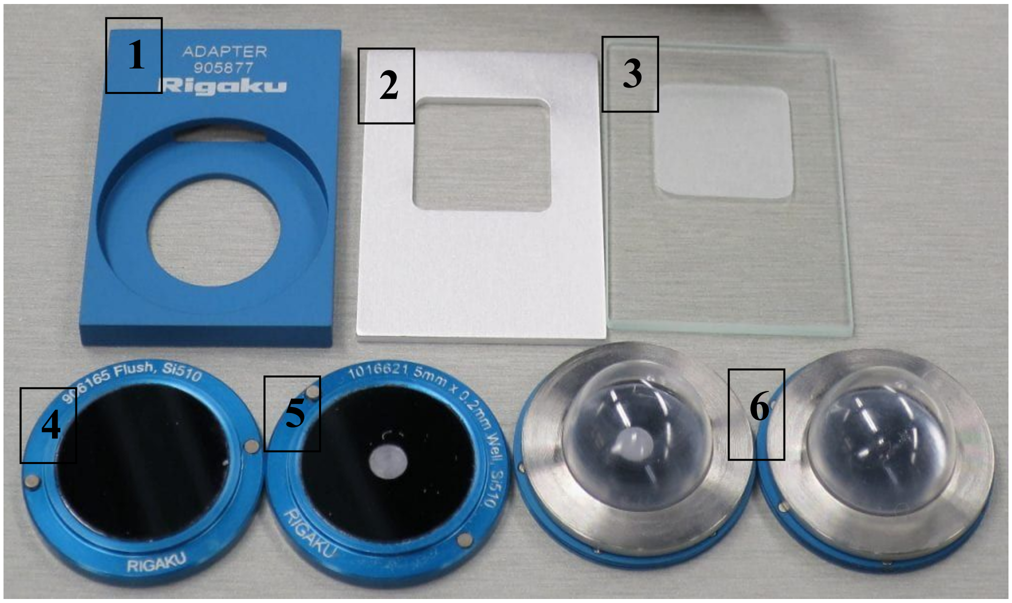


**Figure 5. Different types of sample holders.**

**Steps for Sample Preparation**

Before starting the sample preparation, prioritize safety when handling nanomaterials by working in a certified chemical fume hood to minimize aerosolization and inhalation risks, as nanoparticles can penetrate deep into the lungs or enter the bloodstream due to their small size and high surface area. Wear appropriate personal protective equipment (PPE), including nitrile gloves, safety goggles, a lab coat, and an N95 to guard against skin contact, eye irritation, and respiratory exposure. Avoid generating dust by using anti-static spatulas and antistatic guns for the other used tools, dispose of waste as hazardous nanomaterials per institutional guidelines, and wash hands thoroughly after handling to prevent unintended exposure or surface contamination.

<u>**Sample preparation steps**</u>

- Use finely ground powder samples for analysis. Ensure the particle size is sufficiently small (<200µm) to avoid preferred orientation effects and to maximize random grain orientation.
- Pour the powder sample into the designated glass sample holder or holder tray.
- Distribute the powder evenly and flatten the surface using a flat edge, such as the back of another glass holder or a glass slide. This creates a smooth, level surface parallel to the sample holder, facilitating consistent diffraction angles during measurement.
- Remove any excess powder from the edges or holder surfaces using alcohol-soaked cleaning paper to prevent contamination or scattering artifacts.
- Insert the prepared sample holder into the sample stage inside the instrument chamber. Secure the holder with magnetic clamps to prevent movement during measurement.

- Use care to avoid scratching the sample holder, as scratches can produce unwanted scattering signals. Metal spatulas or sharp tools are not recommended for handling powder samples.

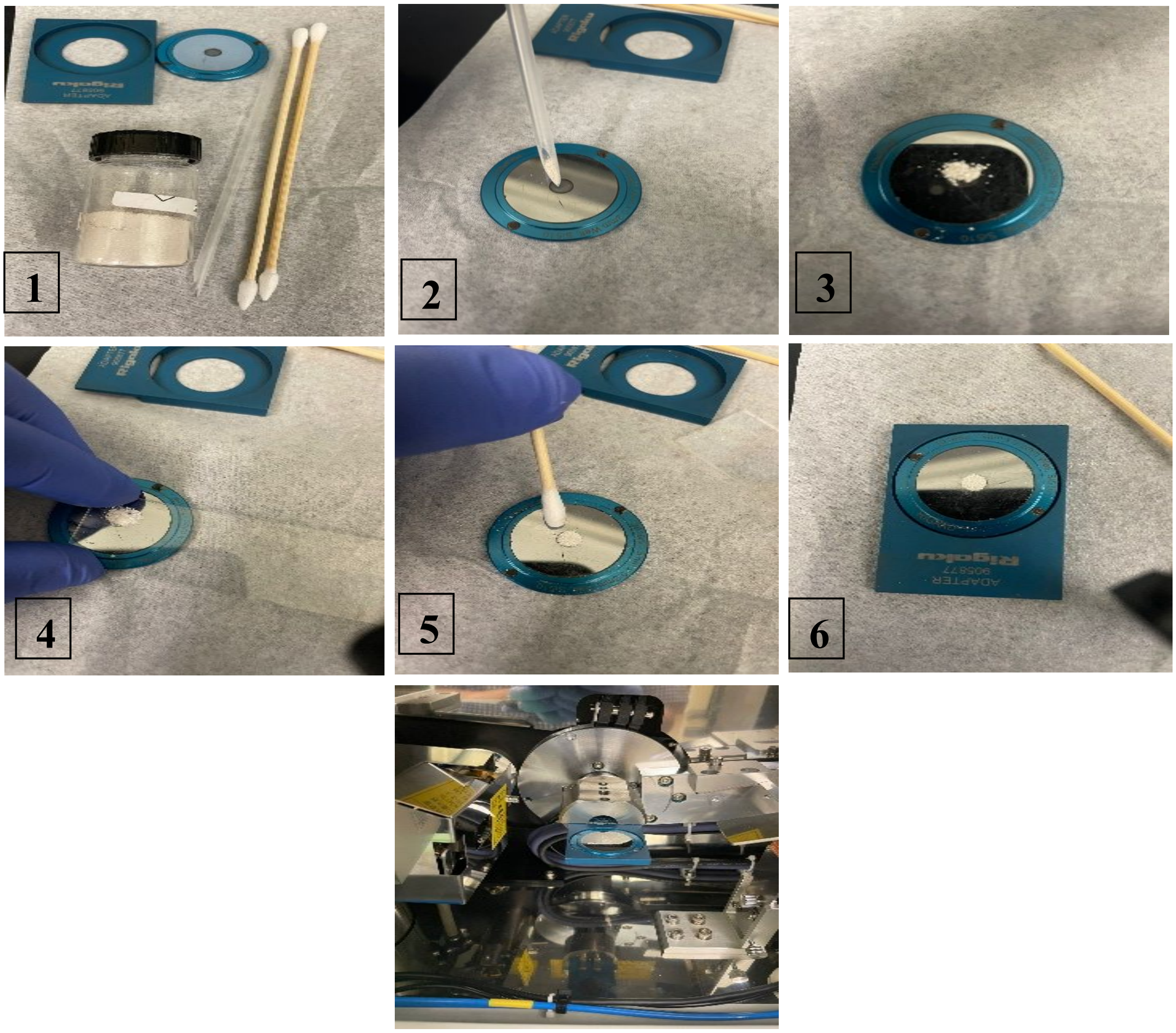


**Figure 6. sample preparation process.**

## Analysis with HighScore Software

In most realistic situations, data analysis is performed on the computer rather than strictly by hand. Many times, a sample's composition is unknown, and powder diffraction is used to gain the information necessary to identify the material. On the terminal connected to the X'PERT, there is a compelling analysis program called HighScore, which greatly aids in this effort. It contains a database with all diffraction data that has ever been published, and can compare the user's data to each entry, producing a list of possible candidates. As an exercise, work through the steps below to see how well HighScore can guess the composition of your samples, and compare your results to those that have been published.

1.Open the program by double-clicking on the icon labeled "X'Pert HighScore," which is located on the desktop.
2.Select FILE->OPEN, and open the XRD file from your experiment. A graph of the data should open on the upper-left of the screen.

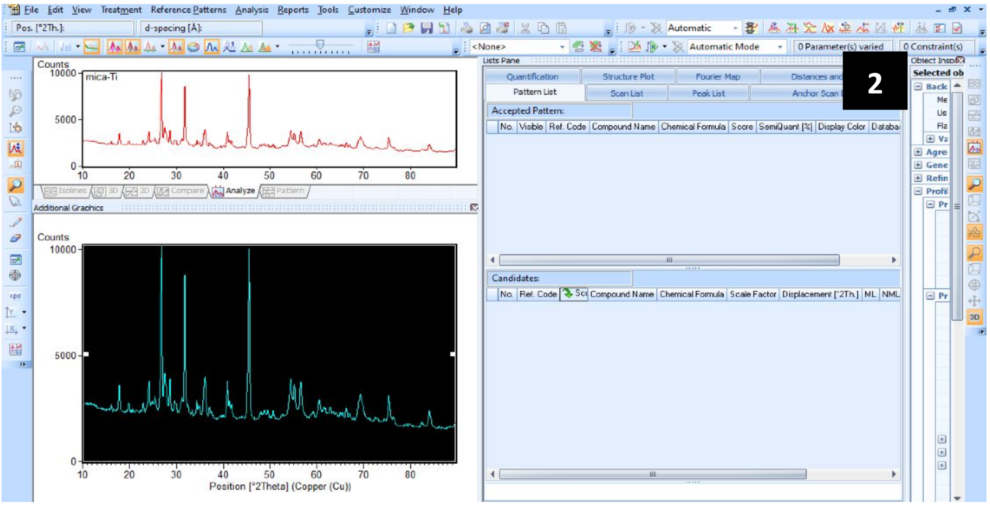


3. In order for HighScore to discern the peaks, you must first specify the background radiation (i.e., the photon count intensity between peaks).

Select TREATMENT>DETERMINE BACKGROUD, which will open up a control window. Keeping your eye on the green line at the bottom of the graph, drag the slider up or down so that it lines up with the background. Once finished, select "Subtract," and then "Replace."

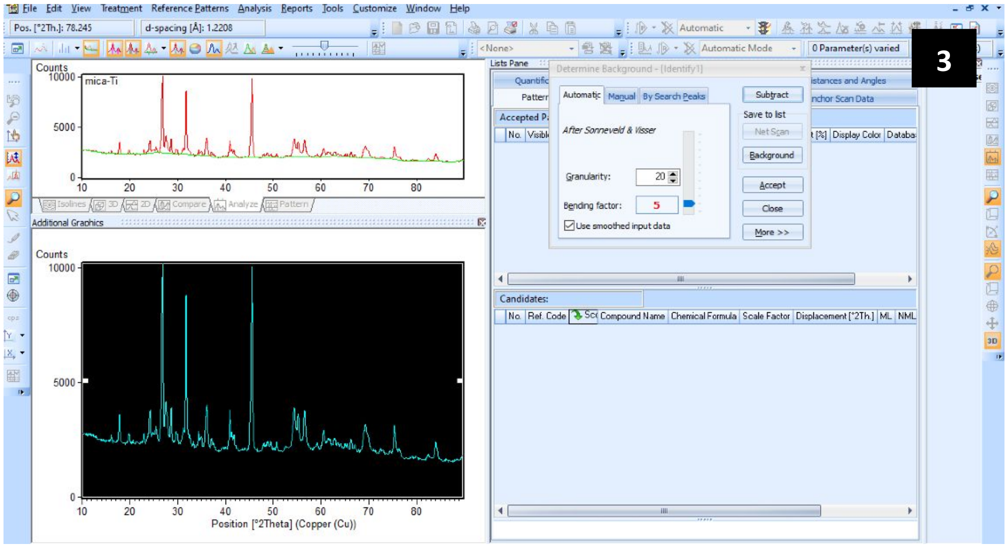


4. Next, we need to have HighScore determine the peak locations that it will compare with the database. To do this, select TREATMENT->SEARCH PEAKS, which will draw red tick marks at all possible peak locations. If it guesses peaks that aren't there, increase the value of "Minimum significance" and click "Search peaks" again; if it doesn't find all of the peaks, then reduce "Minimum significance" and click "Search peaks" until it does. When you are satisfied with the results, click "Replace."

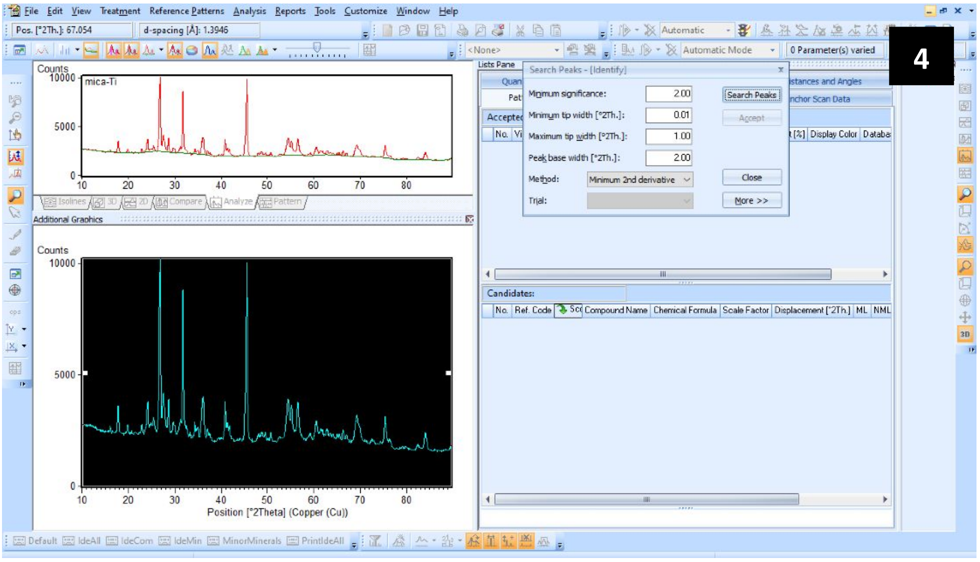


5. Now, we can ask the program to compare the results with the database maintained by the International Center for Diffraction Data (ICDD). Select ANALYSIS>SEARCH & MATCH->EXECUTE SEARCH & MATCH. In the new window, click on the "Restrictions" tab, and select "None." Then, click the "Parameters" tab, and set the data source to "Peak & Profile Data." Finally,

click "Search." 7. At this point, HighScore will give a list of candidates on the right side of the screen, along with a ranking of how well each matches your experiment. By clicking on each search result, the corresponding stick diagram appears in the bottom left window, above that of your data. This indicates only the angles that exactly satisfy Bragg's Law, and shows their relative intensity with respect to each other.

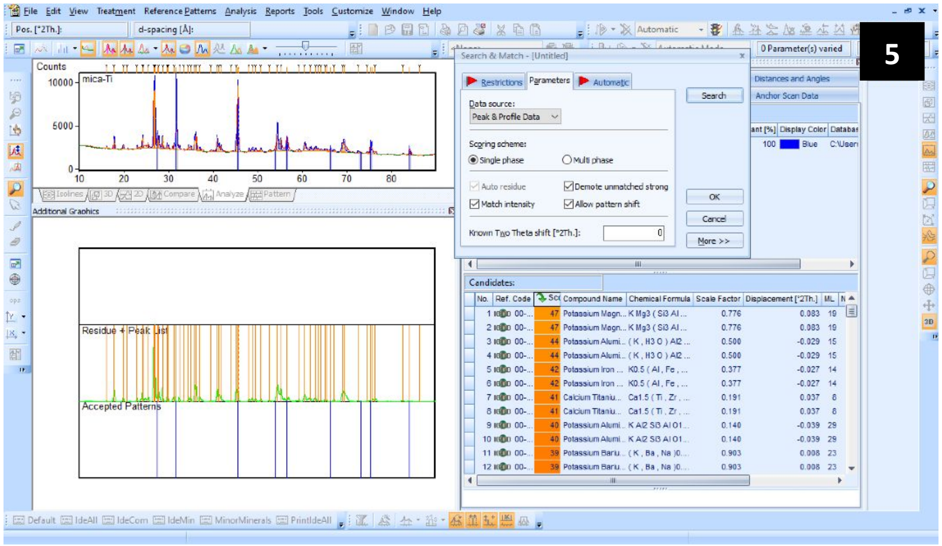


6. For comparison, select REFERENCE PATTERNS->RETRIEVE PATTERN BY>TEXT SEARCH. In the field labeled "Search for String," type in the chemical formula for the sample with a space between each element, and select the bubble labeled "Formula." Then, click "Load," followed by "Close." This will bring up a list of search results in the upper right window double-click any of them to retrieve the published data. You may make a printout of each reference page by copy and pasting into Microsoft Word, or any other text editor on the computer.

## Experimental Work

### Peak and Plane Identification and Pattern Comparison

Each participant group responsible for conducting sample preparation and XRD scans on the materials prepared in Experiments 1 and 2, utilizing guidance from trained operators and following specified instrument parameters.

After running the XRD and obtaining the results data, each group should use HighScore software to identify the characteristic peaks and their corresponding crystallographic planes. "HighScore will help to generate a stick pattern, which illustrates the expected positions and intensities of diffraction peaks based on standard reference data".

To analyze the relationship between the positions (2θ values) and relative intensities of the peaks in the experimental data and the stick pattern, follow these steps:

1. Import standard or reference phase files from the ICCD (International Centre for Diffraction Data) database into HighScore.
2. Overlay the stick pattern of the selected reference phase(s) onto the experimental diffractogram.
3. Assign Miller indices (hkl) to the observed peaks based on the best-matching reference, indicating the diffracting planes present in the sample.

**Safety Notes**

**Sample preparation for XRD analysis**

- Always wear a lab coat, safety goggles, and appropriate gloves; use a dust mask or respirator when working with fine or hazardous powders.
- Handle and weigh powders in a fume hood or dedicated powder hood, using an enclosed balance, and transfer them gently with a spatula rather than pouring to prevent dust clouds.
- Keep sample holders close to the weighing area to shorten transfer distances, wipe any spilled powder immediately with a damp tissue, and dispose of residues in the designated solid-waste container rather than in regular trash.

**Radiation and XRD instrument safety**

- Only authorized users should operate the diffractometer, and all interlocks. Follow all the instructions from the operator while being in the training.

**References**

4. Kiani, D., 2023. X-ray diffraction (XRD). In Springer Handbook of Advanced Catalyst Characterization (pp. 519-539). Cham: Springer International Publishing.
5. Ameh, E.S., 2019. A review of basic crystallography and x-ray diffraction applications. The international journal of advanced manufacturing technology, 105(7), pp.3289-3302.
6. Borchardt-Ott, W., 2011. Crystallography: an introduction. Springer Science & Business Media.
7. Rigaku. "MiniFlex Benchtop X-ray Diffractometer." Rigaku, 2023, https://rigaku.com/products/x-ray-diffraction-and-scattering/xrd/miniflex.

**Post Lab questions**

- What is the main principle behind X-ray diffraction, and why are X-rays specifically used for studying crystal structures?
- Write Bragg's Law and explain each term. How does this law allow one to determine interplanar spacing in crystals from an XRD pattern?
- Describe the features (peak intensity, width, position) of an XRD pattern and what information about the sample each feature provides.
- List the steps taken to identify a crystal structure from a given XRD pattern.
- Why might certain expected peaks be missing from an XRD pattern for a given crystal structure? Give an example.
- How would you distinguish between an amorphous and a crystalline material based on their XRD patterns?

# Experiment 4: Measurement of Magnetic Properties by Vibrating Sample Magnetometry (VSM)

## Aim

Measuring and analyzing the magnetic hysteresis loop of a specific magnetic nanoparticle or composite sample using a Vibrating Sample Magnetometer (VSM) allows for the extraction of key parameters, including saturation magnetization, retentivity, and coercivity.

## Background

Magnetic materials exhibit characteristic responses to applied magnetic fields, summarized by their hysteresis loop a plot of magnetization (M) versus applied magnetic field (H). As the magnetic field increases, domains within a material align, leading to a rise in magnetization until reaching saturation magnetization ($M_s$). If the field is reduced to zero, the material retains some magnetization (remanent magnetization, ($M_r$). To reduce the magnetization to zero, a reverse field called the coercive field ($H_c$) must be applied. The enclosed area of the loop reflects the energy lost in magnetization-demagnetization cycles.

A VSM quantifies these magnetic properties by vibrating a magnetized sample in an external magnetic field and sensing the voltage induced in coils by the changing stray field, as described by Faraday's Law of Induction. The amplitude of this signal is directly proportional to the magnetization of the sample. Sweeping the external magnetic field generates the full hysteresis loop, allowing for extraction of magnetic parameters.

## Hysteresis Loop

The magnetic hysteresis loop is a graph showing the relationship between the applied magnetic field (H) and the magnetization (M) of a ferromagnetic material as the field cycles from positive to negative and back. As the field increases, magnetic domains in the material align, increasing magnetization until saturation magnetization ($M_s$) is reached, where further increase in field causes little change in magnetization. When the applied field is reduced to zero, the material retains some magnetization called remanent magnetization ($M_r$). To demagnetize the sample, a reverse magnetic field equal to the coercivity ($H_c$) must be applied, where magnetization drops to zero. The loop's shape, including saturation, remanence, and coercivity, reveals whether the material is magnetically hard or soft, with hard magnets having large $M_r$ and $H_c$ values. By plotting M versus H from VSM data, students can extract $M_s$ at the plateau regions, $M_r$ at the M-intercept when H=0, and $H_c$ at the H-intercept when M=0, thus fully characterizing the magnetic behavior of their sample.

## General Definitions of Magnetic Characteristics

**Saturation Magnetization ($M_s$):** The maximum magnetization per unit mass (typically in emu/g) achieved when all magnetic moments in a material fully align with an applied magnetic field at sufficiently high strength, representing the intrinsic magnetic content independent of field strength beyond saturation.

**Magnetization Retentivity ($M_r$):** The residual magnetization remaining in a material after the external magnetic field is removed to zero following saturation, resulting from irreversible domain wall pinning and magnetic anisotropy that prevents complete demagnetization.

**Coercivity ($H_c$):** The magnitude of the reverse magnetic field required to bring the magnetization back to zero after the material has been saturated, measuring the material's resistance to changes in its magnetic state due to domain wall motion barriers and spin rotation energies.

These parameters, derived from the hysteresis loop ($M_s$ from plateaus, $M_r$ from M-intercept at H=0, Hc from H-intercept at M=0), classify materials as soft magnets (low $H_c$ < 100 Oe, low $M_r/M_s$ for easy remagnetization in transformers) or hard magnets (high $H_c$ > 1000 Oe, high $M_r/M_s$ for permanent magnets).

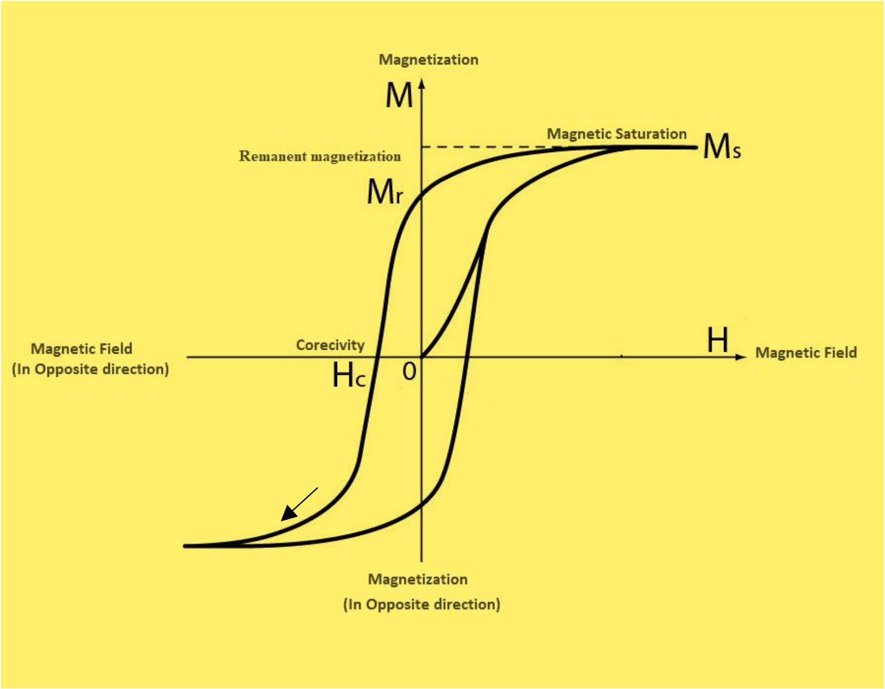


**Figure 1. Hysteresis loop explanation.**

In Figure (2), various magnetic hysteresis loops that represent the magnetic properties of materials classified as soft or hard magnets. The broad loop on the left corresponds to hard magnetic materials, which maintain a significant portion of their magnetization even after the external magnetic field is removed. These materials display high coercivity and remanence, making them suitable for permanent magnet applications where stable and enduring magnetic fields are crucial. While, the middle loop reflects an intermediate magnetic response, characterized by moderate retention and coercivity, typical of materials with mixed or composite magnetic phases. On the right, the narrow loop signifies soft magnetic materials, which exhibit low coercivity and remanence. These materials can be easily magnetized and demagnetized with

minimal energy loss, making them ideal for applications such as transformer cores and electromagnetic circuits that require quick and efficient changes in magnetic fields.

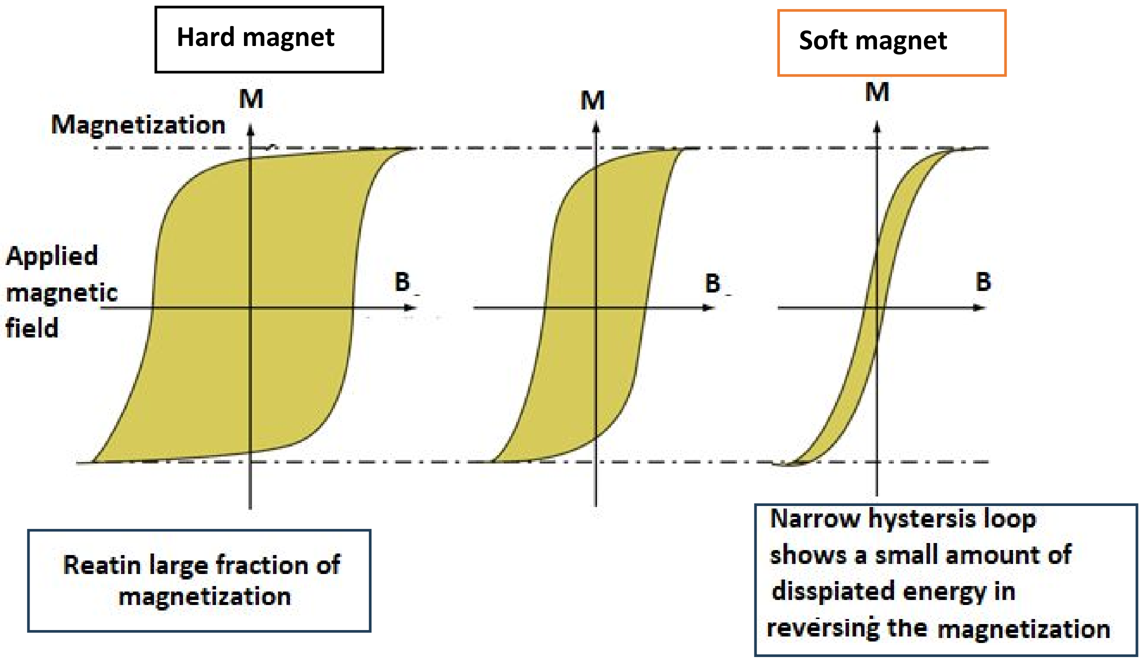


**Figure 2. Variations in Hysteresis loops.**

**Instrumentation of Vibrational Sample Magnetometery**

**<u>The VSM machine setup typically consists of (Figure 4):</u>**

- **Electromagnet:** it produces a strong, uniform magnetic field encompassing the sample.
- **Vibration Exciter and Sample Holder:** Oscillates the mounted sample at a fixed frequency (~85 Hz), ensuring consistent amplitude.
- **Detection/Pick-up Coils:** It surrounds the sample and measures the induced voltage, proportional to the magnetic moment.
- **Lock-in Amplifier and Control System:** Amplify and filter the signal, converting it into digital data connected to analysis software.
- **Computer Interface:** Automates field sweeping, data collection, and plotting of the hysteresis loop.

**<u>Note:</u>** Calibration with a standard magnetic sample (e.g., nickel rod) is usually required to ensure measurement accuracy.

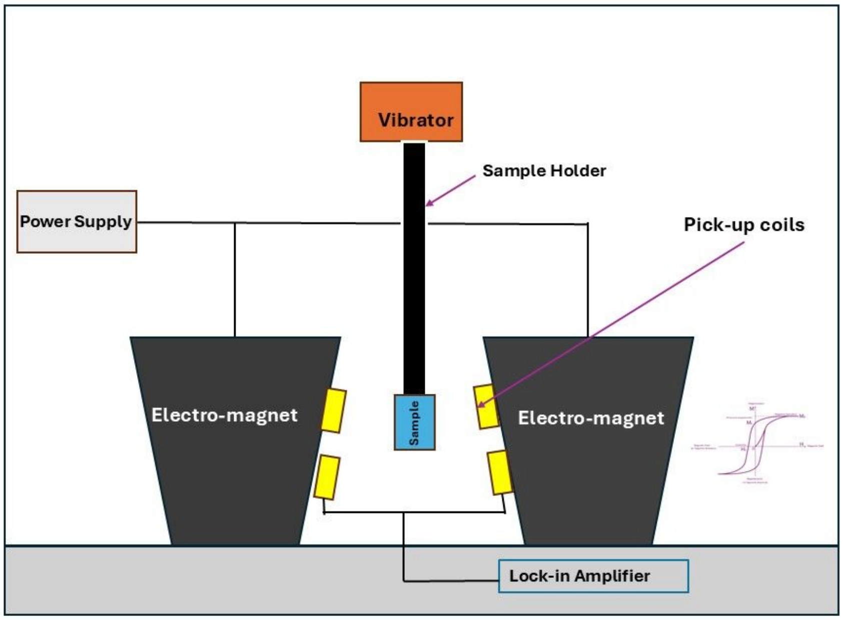


**Figure 3. Illustration of the essential parts of VSM.**

*Shukla, V., 2022. Introduction of Vibrating Sample Magnetometer for Magnetic Characterization. In Handbook of Magnetic Hybrid Nanoalloys and their Nanocomposites (pp. 1-24). Cham: Springer International Publishing.*

**Mechanism of working**

The Vibrating Sample Magnetometer (VSM) operates based on Faraday's Law of Induction, which states that a changing magnetic flux through a coil induces an electromotive force (emf) or voltage. In the VSM setup, the magnetic sample is initially placed in a uniform, constant external magnetic field, generated by an electromagnet, which magnetizes the sample by aligning its magnetic domains.

Subsequently, the sample is mechanically vibrated typically at a frequency of around 80 Hz—perpendicular to the applied magnetic field using a vibration exciter, such as a piezoelectric device. This oscillatory motion causes the magnetic flux from the sample's magnetic dipole moment to vary over time in relation to a set of stationary pick-up coils positioned nearby. As a result of this time-varying flux, an alternating voltage is induced in the pick-up coils, proportional to the sample's magnetization, in accordance with Faraday's Law.

Given that the induced signal is usually quite small, it is amplified using transimpedance amplifiers and lock-in amplifiers to enhance the signal-to-noise ratio. The amplified electrical signal is then digitized and processed using computer software, which correlates the induced voltage to the magnetization of the sample at each external field strength.

By systematically sweeping the magnetic field over a specified range and continuously measuring the sample's response, the VSM provides accurate data on the magnetization curve or hysteresis loop of the material. This enables the extraction of critical magnetic parameters,

including saturation magnetization, remanence, and coercivity, facilitating in-depth analysis of the magnetic properties of the sample.

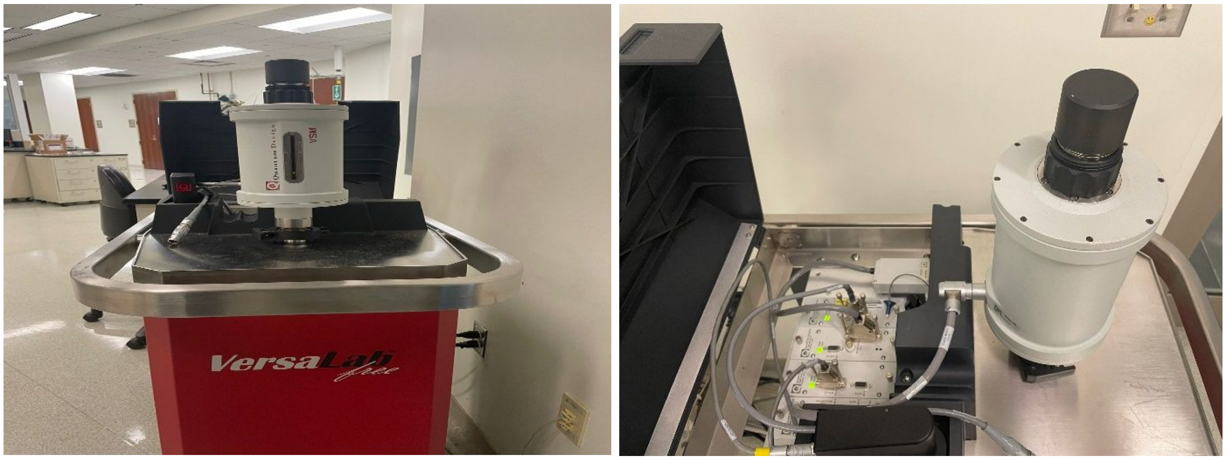


**Figure 4. VSM machine used for magnetic properties evaluation.**

**Experimental Work**

**Sample and instrument preparation for the measurement**

- **System Preparation for VSM Installation**

To prepare the system for the installation of the VSM option, follow these steps using the VSM software:

1. Set the system temperature to 300 K.
2. Adjust the magnetic field to zero (0) Oe.
3. Vent the sample chamber.
4. Remove any sample puck or option currently installed in the sample chamber.

- **Preparing the sample capsule and holder (powder samples)**
  **2.1. Tools needed:**

**Capsules**

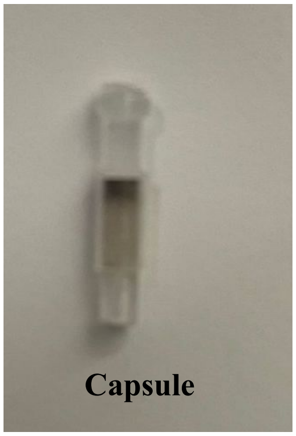
Capsule

Vibrating Sample Magnetometer (VSM) capsules used to hold powder samples are typically made from nonmagnetic materials such as polycarbonate, polypropylene, or quartz, chosen for their low magnetic background and compatibility with cryogenic temperatures. These capsules are usually cylindrical, measuring about 11–15 mm in length and 4.6–5 mm in diameter, and are designed to fit securely within specialized sample holders or brass half-tubes for measurement stability.

***Capsule Loading and preparation***

*These videos illustrating this process are available on the QuantumDesignUSA YouTube channel:* https://www.youtube.com/playlist?list=PLuI5kHh9kg1b2Xc1ZVB5Raq7C-NZYDEuc

**Sample Holder**

Differnent sample holder can be used, the brass half-tube is the common type of sample (capsules) holders which made from cartridge brass tubing with a cobalt-hardened gold plating finish.

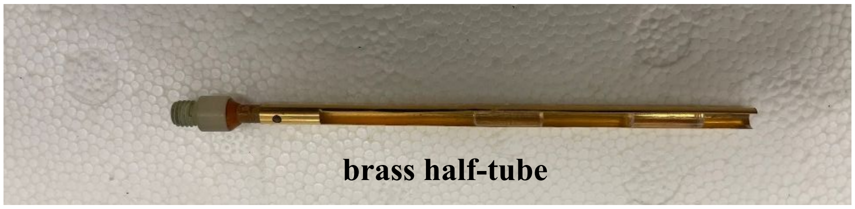
brass half-tube

**Mounting the capsule to the sample holder**

The loading of VSM capsules into the brass sample holder, start by ensuring the powder-filled capsule is clean and tightly sealed, then carefully insert the capsule into the open end of the brass tube so that it sits fully inside the holder, centered both vertically and horizontally, with the capsule tip positioned approximately at the 35 mm reference mark or as specified by the instrument's guidelines for optimal signal detection. Use the mounting station shown in the image to verify that the capsule is aligned within the "Standard" bore groove and does not extend outside the measured sample area, ensuring the capsule is straight and firmly seated to avoid movement during measurements. Handle the assembly with gloves or tweezers to maintain cleanliness, double-check the capsule's alignment relative to the holder's scale, and secure it tightly, as precise positioning within the brass holder is critical for accurate and reproducible VSM results.

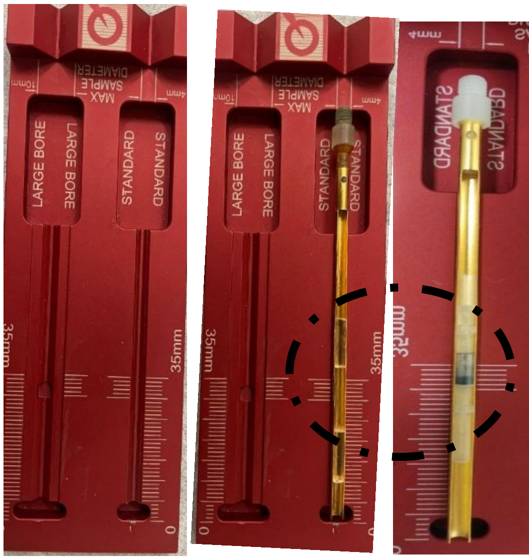

**Mounting Station**

The next step is to attach the assembled holder to the driven sample rod of the VSM system. Secure the brass tube with the capsule into the clamp or attachment fixture at the end of the sample rod, making sure it is tightly fixed and vertically aligned to prevent any wobble or movement during vibration. Always verify that the entire assembly is stable, well-aligned with the instrument axis, and free of loose components before proceeding with measurements. Finally, the rod with the attached sample holder should be inserted inside the machine Inspect the sample rod and sample holder to ensure they are straight. Deviations can result in rubbing of the sample or sample holder in the coil set which causes heating at low temperatures and noise in the measured moment when a magnetic field is applied.

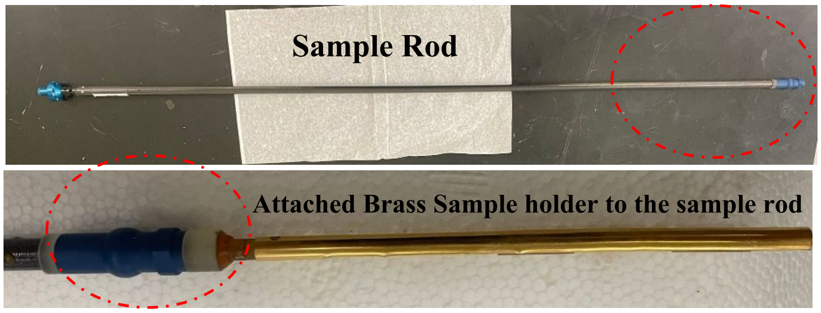


**Final Steps**: Run the sequence as instructed (applied magnetic field and temperature ), once the sequence is complete, remove the components from the brass half-tube. Weigh both pieces, place the powder inside one of the openings, and securely close it with the other piece. Record the total mass of the assembly and track any mass changes in the source.

## References

1- The Editors of Encyclopaedia Britannica. "Hysteresis." Encyclopædia Britannica, 7 Aug. 2025, https://www.britannica.com/science/hysteresis.

2- Paterson, G.A., Zhao, X., Jackson, M. and Heslop, D., 2018. Measuring, processing, and analyzing hysteresis data. *Geochemistry, Geophysics, Geosystems*, *19*(7), pp.1925-1945.

3- Dodrill, B.C., 2015. Measurements with a VSM. *Magnetic Media.–2007*.

4- Dodrill, B. and Lindemuth, J.R., 2021. Vibrating sample magnetometry. In *Magnetic measurement techniques for materials characterization* (pp. 15-37). Cham: Springer International Publishing.

5- Burgei, W., Pechan, M.J. and Jaeger, H., 2003. A simple vibrating sample magnetometer for use in a materials physics course. *American Journal of Physics*, *71*(8), pp.825-828.

6- *Shukla, V., 2022. Introduction of Vibrating Sample Magnetometer for Magnetic Characterization. In Handbook of Magnetic Hybrid Nanoalloys and their Nanocomposites (pp. 1-24). Cham: Springer International Publishing.*

## Post-lab Questions

1. Explain the principle of operation of a Vibrating Sample Magnetometer (VSM). How does it measure the magnetic moment of a sample?
2. Describe the shape of a typical hysteresis loop obtained from a ferromagnetic material using VSM. What does the loop reveal about the magnetic properties of the sample?
3. What is the significance of coercivity and retentivity in the hysteresis loop? How can these be determined from the loop?
4. How does the hysteresis loop differ between soft and hard magnetic materials as observed in a VSM experiment? Give examples.
5. What factors can affect the accuracy and quality of hysteresis loop measurements in a VSM? How can these be minimized?

**REU Four-Week Experiment Presentation Guide**

After completing the four-week REU experiments, as part of the research experience, each group is required to prepare an effective presentation to communicate the research and the experiments performed. The presentation should begin with a concise introduction that outlines the research objectives and the significance of your project. Follow this with a brief overview of the experimental methods used, emphasizing key techniques such as X-ray Diffraction (XRD) and Vibrating Sample Magnetometry (VSM). All data obtained from these instruments must be presented visually through clear, well-organized plots and graphs. These should highlight essential features such as peak positions, intensity patterns in XRD, or magnetic hysteresis loops from VSM. Each figure should be accompanied by a detailed interpretation explaining the physical or material properties revealed by the data, linking observations back to the research questions. To enhance engagement and clarity, include high-quality photos of your experimental setup and procedures, labeled appropriately to illustrate the work process. Structure your talk with distinct sections for results, discussion, and a conclusion that summarizes key findings and suggests potential future work or applications.

Pay attention to slide design by minimizing text, using legible fonts, and ensuring all visuals are clear from a distance. Practice your delivery to maintain a steady pace, speak clearly, and be prepared to answer audience questions thoughtfully. This approach will not only showcase the depth of your research but also your ability to communicate scientific information effectively, honoring the effort invested throughout the REU program and leaving a strong impression on your audience.
